# Lamin A/C and Emerin depletion impacts chromatin organization and dynamics in the interphase nucleus

**DOI:** 10.1186/s12860-019-0192-5

**Published:** 2019-05-22

**Authors:** Devika Ranade, Roopali Pradhan, Muhunden Jayakrishnan, Sushmitha Hegde, Kundan Sengupta

**Affiliations:** Indian Institute of Science Education and Research (IISER)-Pune, Dr. Homi Bhabha Road, Biology, Room#B-216, 1st Floor, Main Building, Pashan, Pune, Maharashtra 411008 India

**Keywords:** Nucleus, Chromosome territories, Lamin A/C, Emerin, Nuclear myosin 1, Actin

## Abstract

**Background:**

Nuclear lamins are type V intermediate filament proteins that maintain nuclear structure and function. Furthermore, Emerin - an interactor of Lamin A/C, facilitates crosstalk between the cytoskeleton and the nucleus as it also interacts with actin and Nuclear Myosin 1 (NM1).

**Results:**

Here we show that the depletion of Lamin A/C or Emerin, alters the localization of the nuclear motor protein - Nuclear Myosin 1 (NM1) that manifests as an increase in NM1 foci in the nucleus and are rescued to basal levels upon the combined knockdown of Lamin A/C and Emerin. Furthermore, Lamin A/C-Emerin co-depletion destabilizes cytoskeletal organization as it increases actin stress fibers. This further impinges on nuclear organization, as it enhances chromatin mobility more toward the nuclear interior in Lamin A/C-Emerin co-depleted cells. This enhanced chromatin mobility was restored to basal levels either upon inhibition of Nuclear Myosin 1 (NM1) activity or actin depolymerization. In addition, the combined loss of Lamin A/C and Emerin alters the otherwise highly conserved spatial positions of chromosome territories. Furthermore, knockdown of Lamin A/C or Lamin A/C-Emerin combined, deregulates expression levels of a candidate subset of genes. Amongst these genes, both KLK10 (Chr.19, Lamina Associated Domain (LAD+)) and MADH2 (Chr.18, LAD-) were significantly repressed, while BCL2L12 (Chr.19, LAD-) is de-repressed. These genes differentially reposition with respect to the nuclear envelope.

**Conclusions:**

Taken together, these studies underscore a remarkable interplay between Lamin A/C and Emerin in modulating cytoskeletal organization of actin and NM1 that impinges on chromatin dynamics and function in the interphase nucleus.

**Electronic supplementary material:**

The online version of this article (10.1186/s12860-019-0192-5) contains supplementary material, which is available to authorized users.

## Background

Chromosomes are organized as Chromosomes Territories (CT) in the interphase nucleus. Gene rich chromosome territories are positioned near the nuclear interior, while gene poor chromosome territories are proximal to the nuclear periphery [1, 2]. Fluorescence microscopy and contact based chromatin maps such as Chromosome Conformation Capture (3C) and Hi-C further reiterate the non-random organization of the genome [1–4].

Lamins are type V intermediate filament proteins, enriched at the nuclear envelope that regulate nuclear structure-function relationships and are required for maintaining the non-random organization of chromosome territories [5]. Lamin A and Lamin C are encoded by the LMNA gene, while B type lamins (Lamin B1 and Lamin B2) are encoded by two different genes - LMNB1 and LMNB2 [6, 7]. Lamins A, C, B1 and B2 form separate but interacting microdomains in the nucleus [5, 8]. While B type lamins are expressed in all cells, Lamin A/C expression is lower in stem cells and increases as cells differentiate [9–12]. In addition to their localization and abundance at the nuclear envelope, a sub-fraction of both A and B type Lamins also exist in the nucleoplasm [13–20]. Furthermore, Lamins are also involved in transcription, replication, DNA repair and senescence [21–24]. Lamins interact with chromatin either directly [25–28] or indirectly through chromatin binding proteins. B type lamins interact with Lamin B Receptor (LBR) and HP1α associated with heterochromatin [29], while A type lamins interact with proteins associated with both hetero and euchromatin namely LAP2α, Emerin and BANF1 [30–32]. A and B type lamins along with their interactors such as Emerin and LAP2β (Lamina associated polypeptide 2β) associate with chromatin at ‘Lamina Associated Domains (LADs)’ [33–35]. LADs are ~ 0.1–10 Mb stretches of chromatin bearing Lamina Associated Sequences (LASs), characterized by high density of repeat rich DNA and inactive histone marks H3K9me2/3 [33, 36, 37]. LADs bound by B type lamins are proximal to the nuclear envelope, while those bound by A type lamins exist both at the nuclear periphery and interior respectively [38, 39].

The functional dichotomy between the sub-interactomes of Lamins at the nuclear interior (Lamin A/C with LAP2α, BANF1) and nuclear periphery (Lamin A/C with Emerin, SUN1, SUN2, Nesprin-1α, Nesprin-2 and B-type Lamins with LBR, HP1α) is unclear [29–32, 40–45]. The contrasting organization of Lamin A at the nuclear envelope as a meshwork, and the nuclear interior as foci, short fibrous structures or unstructured nucleoplasmic ‘veil’ suggests differential associations of Lamin A with its interactors, resulting in distinct downstream effects on chromatin organization and function [8, 16, 20, 46–51]. Interestingly, in stem cells depleted of all Lamins, LAD organization is maintained by Emerin - a member of the LEM-D protein family, that interacts with Lamin A/C at the nuclear envelope [52].

Mutations in Lamin A/C and Emerin are associated with muscular dystrophies, suggestive of cell type specific co-regulation in Lamin A/C and Emerin function [53]. Lamin A mutations associated with cardiomyopathy (E161K) and progeria (G608G) show aberrant chromosome positions and gene expression profiles [54–57]. Additionally, patient-derived fibroblasts with laminopathy mutations (R89L, E358K, R482L in LMNA) and X-EDMD fibroblasts (ED5364, with mutations in EMD) reposition CT13 and CT18 (gene poor) toward the nuclear interior [58]. Emerin is localized at the interface of the cytoplasm and the nucleus, and functions as a mechanosensitive protein which regulates the response of the genome to external mechanical stimuli [59–61]. Furthermore, Emerin and Lamin A/C bind to actin and nuclear myosin 1 [62, 63]. Nuclear myosin 1 (NM1) is required for chromatin remodeling during transcription by RNA Polymerase I, II and III and functions in actin assisted long range chromatin movements [64–67]. A complex involving Lamin A/C-Emerin-NM1 along with actin, modulates chromatin dynamics [62, 68–72]. Emerin stabilizes the growing ends of F-actin, while Lamin A/C binds to and regulates the levels of G-actin in the nucleus [62, 63, 73–75].

Here we show that the loss of Lamin A/C or Emerin alters cytoskeletal organization with an increase in nuclear NM1 foci and actin stress fiber aggregates. Loss of Lamin A/C and Emerin, further alters chromosome territory positions and enhances chromatin mobility, which was rescued to basal levels upon NM1 inhibition or actin depolymerization. Remarkably, Lamin A/C and Emerin alters expression levels of genes, which further show differential association with the nuclear envelope. In summary, these results underscore a remarkable role for Lamin A/C and Emerin as modulators of cytoskeletal and chromatin organization in the interphase nucleus.

## Results

### Lamin A/C or Emerin knockdown increases nuclear myosin 1 (NM1) foci in the nucleus

**Fig. 1:**
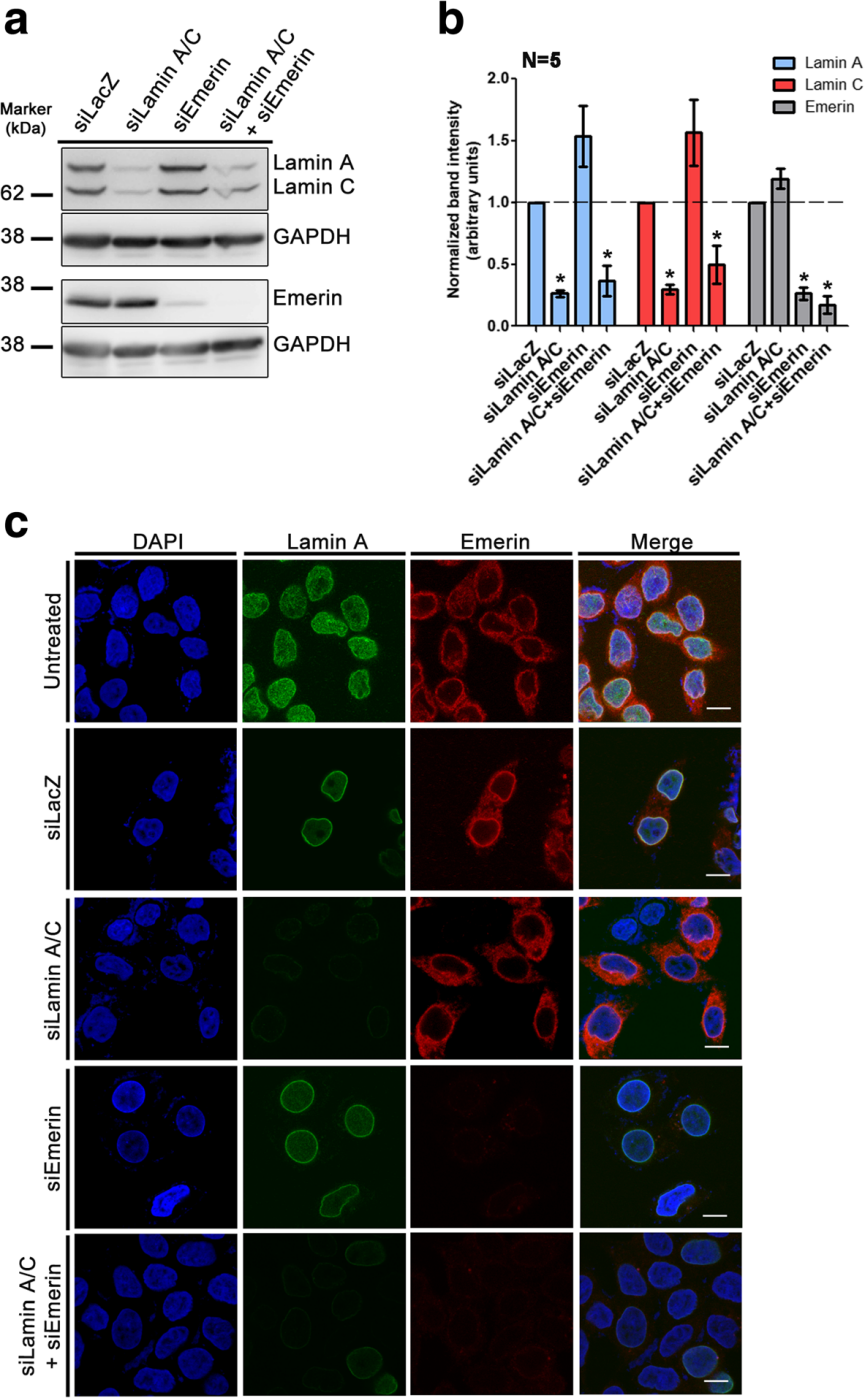
Lamin A/C and Emerin knockdowns in DLD-1 cells. **a** Representative western blot showing single or combined knockdowns of Lamin A/C, Emerin in DLD-1 cells. Control: non-targeting siRNA (siLacZ), Loading control: GAPDH. **b** Densitometric quantification of band intensities for Lamins A, C and Emerin (*N* = 5 independent biological replicates, *p < 0.05). This shows ~ 75–80% knockdown of Lamin A/C and Emerin in both single and co-depletions respectively **c** Immunostaining of Lamin A (green) and Emerin (red) in single and co-depleted DLD-1 cells. Scale bar ~ 10 μm, (*N* = 2, data compiled from two independent biological replicates (N))

Emerin is localized in the cytoplasm and at the nuclear envelope of cells [76, 77]. Lamin A/C and Emerin are part of a nuclear sub-complex, that partner with Nuclear Myosin 1 (NM1) and actin [62, 63, 73]. NM1 and actin independently modulate chromatin dynamics and long range chromatin movements [66, 67, 78]. Furthermore, NM1 associates with chromatin, while G-actin assists chromatin remodeling in gene rich regions, predominantly toward the nuclear interior [65, 68, 79, 80]. We sought to investigate the impact of single and combined knockdowns (Kd) of Lamin A/C and its interacting partner – Emerin, on the subcellular localization of NM1 and actin in DLD-1 cells. Both single and combined knockdowns, depleted Lamin A/C and Emerin to ~ 70%, as ascertained independently by immunoblotting and immunofluorescence assays respectively (Fig. 1a-c).

**Fig. 2:**
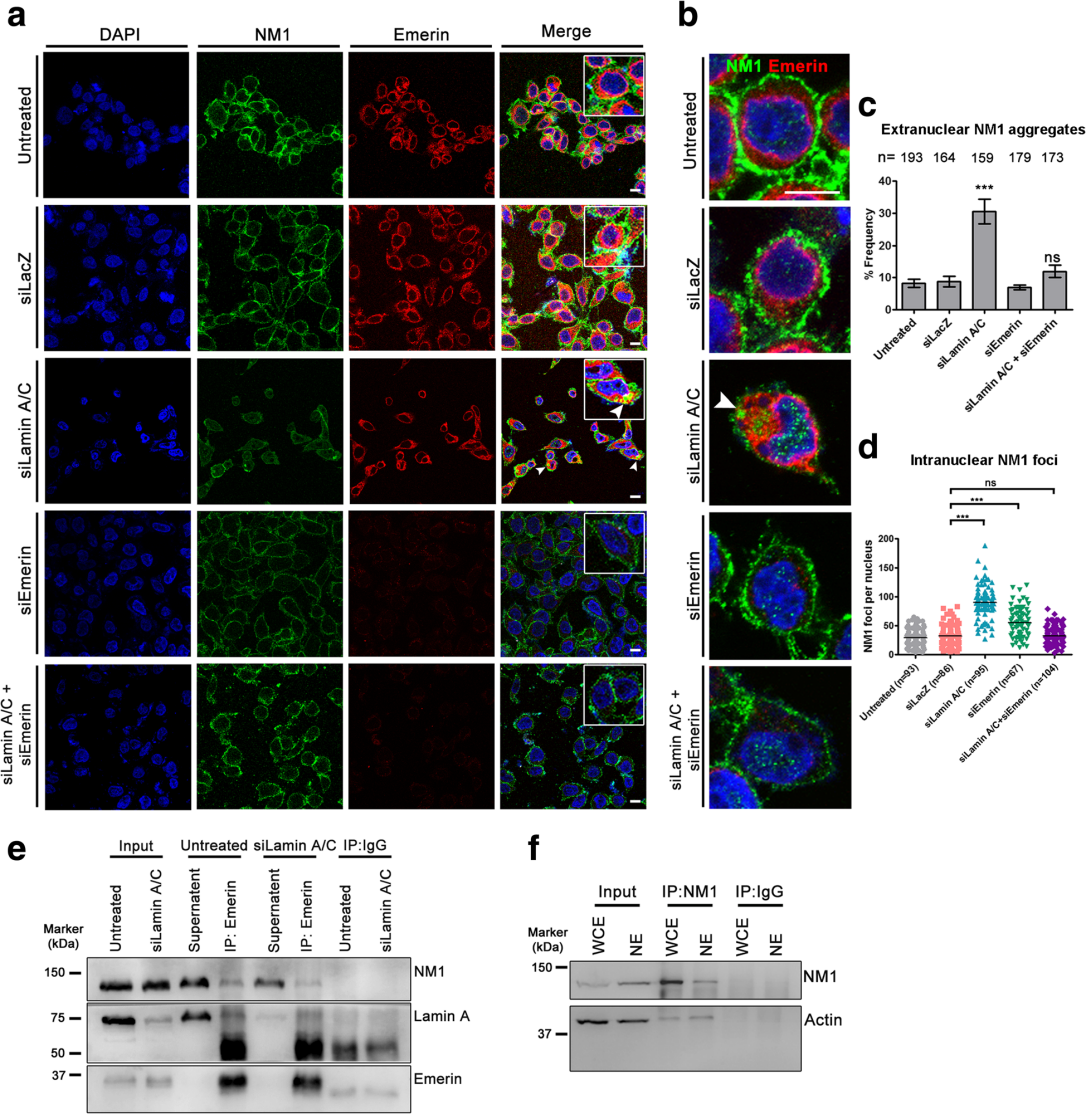
Lamin A/C depletion mislocalizes nuclear myosin I (NM1) in an Emerin dependent manner. **a** Immunostaining for NM1 (green) and Emerin (red) in control (untreated, siLacZ), single and combined knockdowns of Lamin A/C and Emerin. Scale bar ~ 10 μm. Inset: Zoomed in images **b** Single representative nuclei showing intranuclear NM1 foci (green) and Emerin (red) in control (untreated, siLacZ) cells, single and co-depletion of Lamin A/C and Emerin. Scale bar ~ 10 μm **c** Mean % frequency of cells (from **a**, **b**) showing aggregates of NM1 in the cytoplasm. Error bars: SEM, *N* = 3, ***p < 0.0001 **d** Dot scatter plot showing number of intranuclear NM1 foci (mid-optical section) in control (untreated, siLacZ) cells, single and co-depletion of Lamin A/C and Emerin. Horizontal lines: Median (M), n = number of nuclei analysed, N = 2, *** p < 0.0001 (N: number of independent biological replicates) **e** Representative Co-IP using anti-Emerin antibody, probed for NM1 and Lamin A in control (untreated) and Lamin A/C depleted cells (48 h post Lamin A/C Kd, N = 3) **f** Co-IP performed on whole cell extracts (WCE) and nuclear extracts (NE) of DLD-1 cells using anti-NM1 antibody and probed for actin (N = 2)

We examined the subcellular localization of NM1 upon single and co-depletion of Lamin A/C and Emerin by immunofluorescence staining (Fig. 2a-b). NM1 is typically localized at the plasma membrane as a dense fibrous network and marginally in the nucleoplasm as punctate foci [81] (Fig. 2a-b, untreated, siLacZ). As reported previously, Lamin A/C knockdown results in an extranuclear aggregation of Emerin (Additional file 1a-b) [82]. Interestingly, Lamin A/C Kd alone, also showed a significant increase in NM1 aggregates outside the nucleus (Fig. 2b-c). Additionally, the number of intranuclear NM1 foci showed a significant increase upon both the single depletions of Lamin A/C and Emerin (Fig. 2d, Additional file 1c). Remarkably, NM1 localization was unaltered in the Lamin A/C and Emerin co-depleted cells (Fig. 2c-d, Additional file 1c). Immunoblotting did not show a significant change in the total levels of NM1 in either single or co-depletions of Lamin A/C and Emerin (Additional file 2a-b). Taken together, this suggests that Lamin A/C and Emerin modulate the nuclear localization of NM1.

Consistent with previous studies, co-immunoprecipitation assays showed that Lamin A/C interacts with Emerin and Emerin is a known interactor of NM1 (Fig. 2e) [30, 62]. Furthermore, Lamin A/C knockdown, reduces the association between Emerin and NM1, suggesting that Lamin A/C stabilizes Emerin-NM1 interaction (Fig. 2e, Additional file 2c-d). Independent co-immunoprecipitation assays also revealed an association between NM1 and actin in the nucleus (Fig. 2f) [83]. In summary, these results implicate Lamin A/C as a modulator of NM1 localization in cells, as it regulates the stability of Emerin-NM1 interactions.

### Lamin A/C-Emerin loss enhances actin stress fibers in cells

Lamin A/C and Emerin independently modulate actin polymerization in cells [62, 63, 73]. The arrangement of F-actin filaments impacts cell morphology, contractility and cell mechanics [84]. Cells on micropatterned substrates show increased actin polymerization and decreased diffusion of labelled H2B, suggesting an impact of altered actin organization on the nucleus and chromatin dynamics [69].

**Fig. 3:**
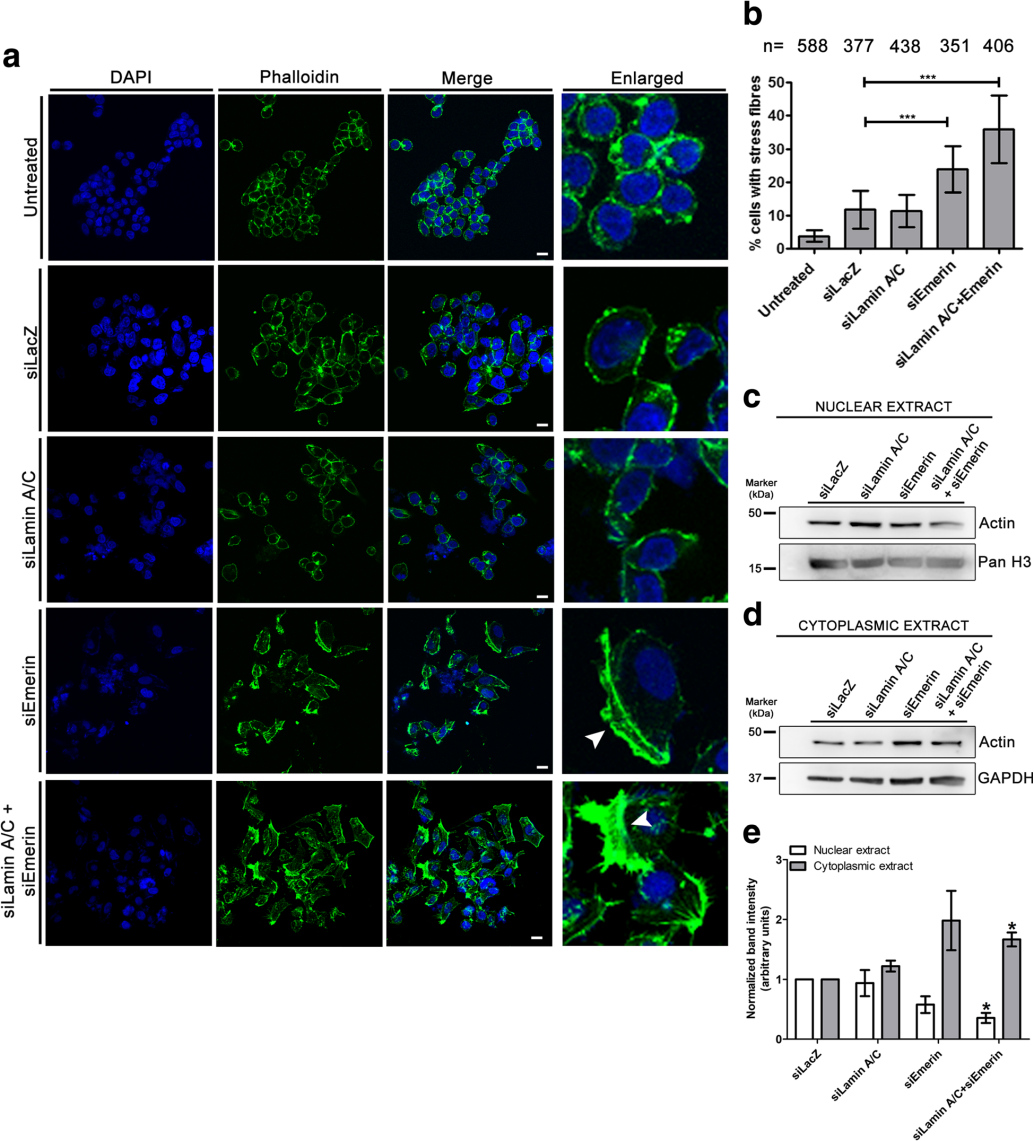
Increase in actin stress fiber aggregates upon Lamin A/C-Emerin co-depletion. **a** Phalloidin staining (green) in control (untreated and siLacZ) cells, and upon single and co-depletion of Lamin A/C and Emerin. Scale bar ~ 10 μm. Enlarged: Zoomed in images. **b** % of cells with actin aggregates (from a), n = number of cells analysed, Error bars: SEM, N = 3, ***p < 0.0001 (N: number of independent biological replicates). **c** Representative western blot showing Actin levels in nuclear extracts of DLD-1 cells with single and combined depletion of Lamin A/C-Emerin **d** Representative western blot showing Actin levels in cytoplasmic extract of DLD-1 cells upon single and combined depletion of Lamin A/C-Emerin **e** Densitometric quantification of band intensity for Actin levels in nuclear and cytoplasmic extracts from cells with single and combined depletion of Lamin A/C-Emerin. Error bar: SEM, data compiled from N = 3 independent biological replicates, *p < 0.05

We stained untreated DLD-1 cells with Phalloidin, which shows a distinct cobblestone morphology characteristic of epithelial cells, with relatively fewer stress fibers (Fig. 3a). Lamin A/C depletion alone did not appreciably alter actin stress fibers (Fig. 3a-b). In contrast, Emerin knockdown showed a significant increase in cells with actin stress fibers (~ 23%, arrowheads, Fig. 3a-b). Combined knockdown of Lamin A/C and Emerin also increased actin stress fibers in cells (~ 35%, arrowheads, Fig. 3a-b). Interestingly, we observed a decrease in actin levels in the nucleus as compared to the cytoplasm, upon immunoblotting nuclear and cytoplasmic extracts prepared from Emerin and Lamin A/C-Emerin co-depleted cells (Fig. 3c-e, Additional file 3c-d). Taken together, Lamin A/C and Emerin modulate subcellular distribution and organization of actin in cells.

### Lamin A/C and Emerin co-depletion mislocalizes chromosome territory positions

Lamin A/C interacts with Emerin at the inner nuclear membrane [30]. Lamin A/C and Emerin regulate nuclear shape, mechanosignaling and chromatin organization via their common interactor - Barrier to Autointegration Factor (BAF) [30, 75, 85]. We previously showed that loss of Lamin A/C alone, does not alter the spatial positions of chromosome 18 and 19 territories in diploid DLD-1 cells [86].

**Fig. 4:**
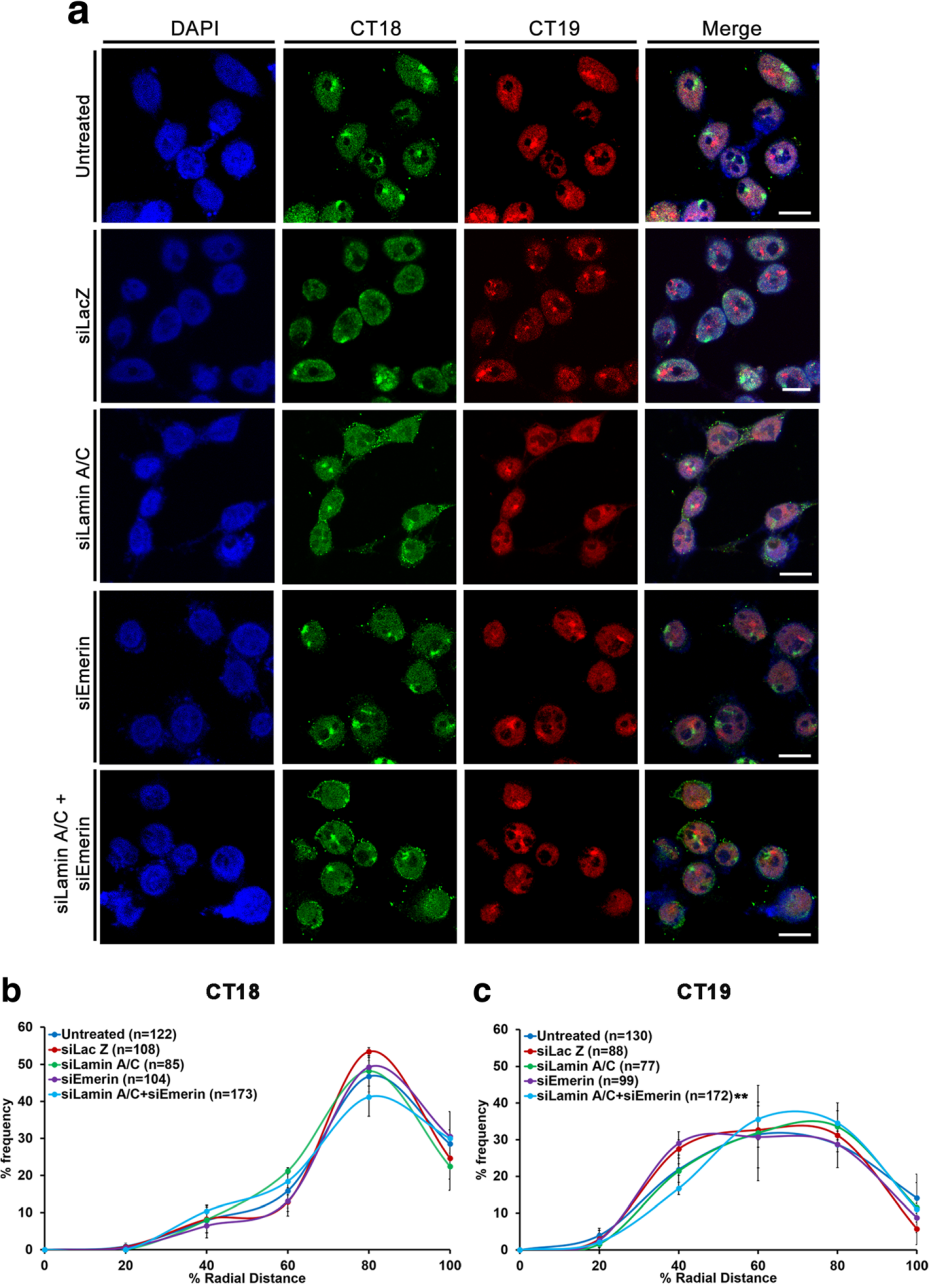
Lamin A/C-Emerin co-depletion mislocalizes chromosome 19 territories. **a** Representative 3D-FISH images for CT18 (green) and CT19 (red) in control (untreated and siLacZ) cells, and single and co-depletion of Lamin A/C and Emerin. Scale bar ~ 10 μm. **b-c** Radial Distance (% R.D) distribution profiles for (**b**) CT18 and (**c**) CT19 in control (untreated and siLacZ) cells, and single and co-depletion of Lamin A/C and Emerin (X-axis: 0%: nuclear centre, 100%: nuclear periphery). Error bar: SEM, ** *p* < 0.01, n = number of CTs analysed, data compiled from N = 3 (N: number of independent biological replicates)

We asked if the co-depletion of Lamin A/C and Emerin perturbs chromosome territory positions in DLD-1 cells (Additional file 3a). We examined positions of chromosome 18 and 19 territories since CT18 (Gene Density ~ 12.35 genes/Mbp, Size: 80 Mbp) and CT19 (Gene Density ~ 42.05 genes/Mbp, Size: 59 Mbp) are of comparable DNA content but strikingly contrasting gene densities (Fig. 4a) [1, 87, 88]. Lamin A/C or Emerin knockdowns did not alter the conserved spatial positions of either CT18 (peripheral) or CT19 (internal) in the nucleus (Fig. 4b-c, Table 1). Remarkably, co-depletion of Lamin A/C and Emerin significantly repositioned gene rich CT19 away from the nuclear interior (Median (M) = 59.57% R.D, Control – siLacZ: M = 53.35% R.D) (Fig. 4c, Table 1). However, the otherwise peripheral nuclear location of CT18 was unaltered upon the co-depletion of Lamin A/C and Emerin (M = 75.46% R.D, Control - siLacZ: M = 74.85% R.D) (Fig. 4b, Table 1), despite the spatial proximity of gene poor CT18 to the nuclear envelope.

**Table 1:** Median % Radial Distance of CT18 and CT19 upon co-depletion of Lamin A/C and Emerin

Chromosome/Gene Density	Experiment	%Radial Distance (R.D)
CT18 (~ 12.35 genes/Mbp)	Untreated	72.52
	siLacZ	74.85
	siLamin A/C	71.17
	siEmerin	75.06
	siLamin A/C + siEmerin	75.46
CT19 (~ 42.05 genes/Mbp)	Untreated	53.52
	siLacZ	53.35
	siLamin A/C	51.65
	siEmerin	50.72
	siLamin A/C + siEmerin	59.57^a^ (*p* = 0.0091)

^a^: statistically significant

**Fig. 5:**
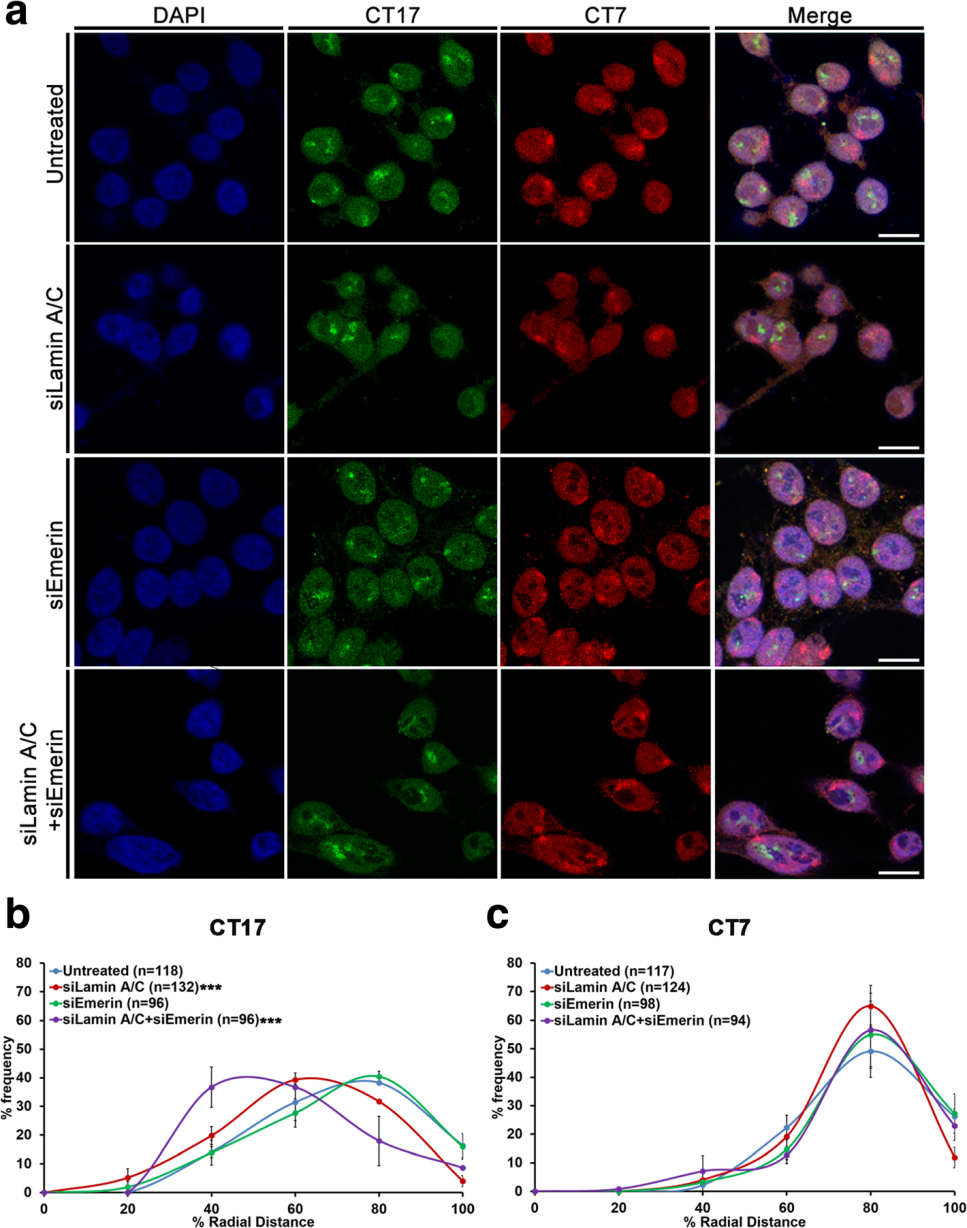
Lamin A/C-Emerin co-depletion mislocalizes chromosome 17 territories. **a** Representative 3D-FISH images for CT7 (red) and CT17 (green) in control (untreated) cells, and single and co-depletion of Lamin A/C and Emerin. Scale bar ~ 10 μm. **b-c** Radial Distance (% R.D) distribution profiles for (**b**) CT17 and (**c**) CT7 in control (untreated) cells, and single and co-depletion of Lamin A/C and Emerin (X-axis: 0%: nuclear centre, 100%: nuclear periphery). Error bar: SEM, *** *p* < 0.001, n = number of CTs analysed, compiled data from N = 2 (N: number of independent biological replicates)

We next determined the positions of another pair of chromosome territories i.e. gene rich CT17 (Gene Density ~ 29.3 genes/Mbp) and gene poor CT7 (Gene Density ~ 17.45 genes/Mbp) (Fig. 5a). Gene rich CT17 succeeds CT19 in gene density. The gene rich CT17 shifted more toward the nuclear interior upon Lamin A/C Kd (M = 53.42% R.D, Control - Untreated: M = 61.43% R.D) and in Lamin A/C-Emerin co-depleted cells (M = 50.27% R.D) (Fig. 5a-b, Table 2). However, similar to gene poor CT18, the positions of gene poor CT7 (peripheral), remained unaltered in either single or combined knockdowns of Lamin A/C and Emerin (Fig. 5a, c, Table 2). Consistent with previous findings, Lamin A/C knockdown also affects the topology of chromosome territories (Additional file 4a-j) [56]. In summary, Lamin A/C and Emerin play a combined role in maintaining the conserved positions of chromosome territories present away from the nuclear envelope in the interphase nucleus.

**Table 2:** Median % Radial Distance of CT 7 and CT17 upon co-depletion of Lamin A/C and Emerin in DLD-1 cells

Chromosome/Gene Density	Experiment	%Radial Distance (R.D)
CT7 (17.45 genes/Mbp)	Untreated	71.69
	siLamin A/C	68.87
	siEmerin	73.35
	siLamin A/C + siEmerin	71.20
CT17 (29.30 genes/Mbp)	Untreated	61.43
	siLamin A/C	53.42^a^ (*p* = 0.0202)
	siEmerin	62.00
	siLamin A/C + siEmerin	50.27^a^ (*p* = 0.0137)

^a^: statistically significant

### Lamin A/C and Emerin co-depletion enhances chromatin mobility

**Fig. 6:**
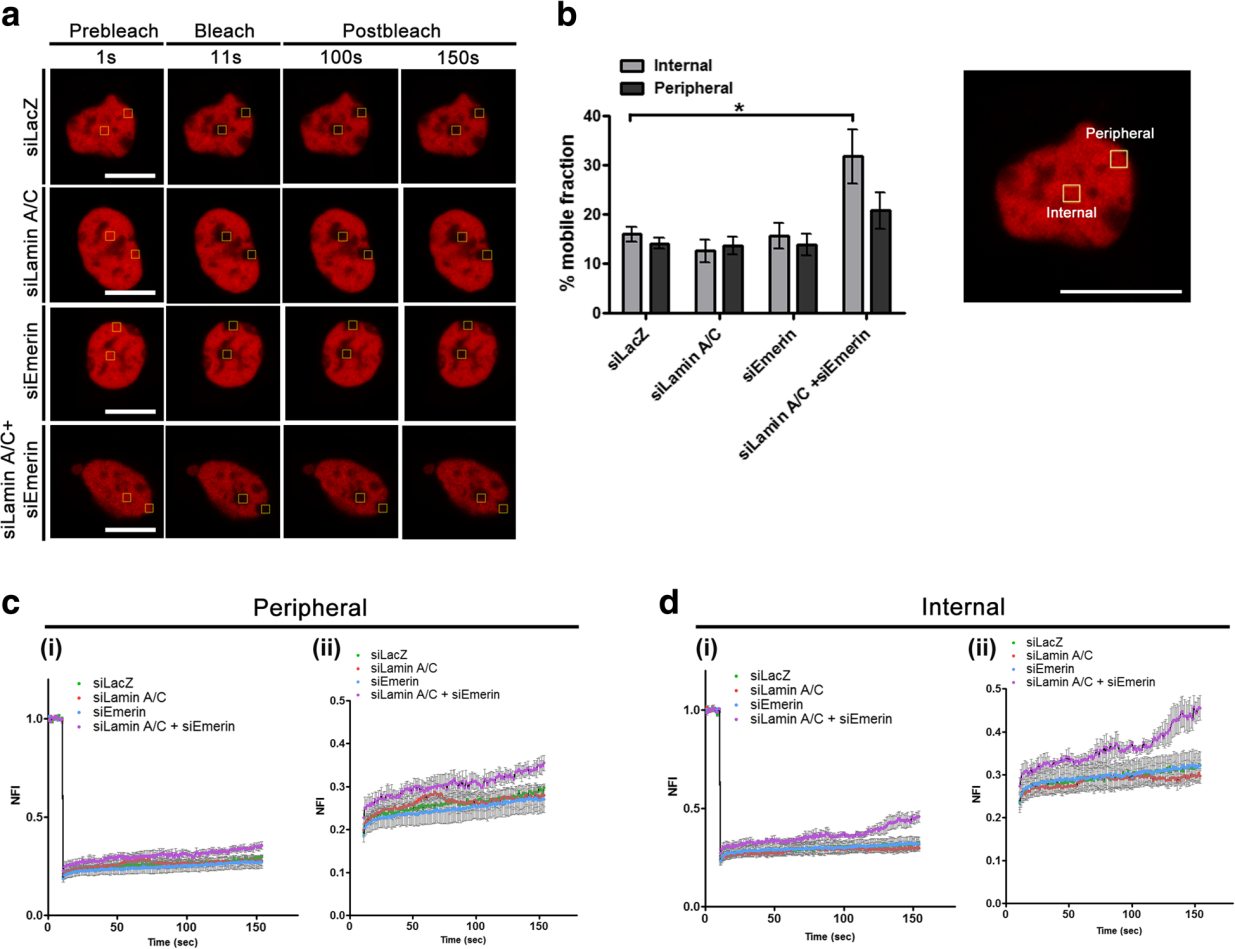
Lamin A/C-Emerin co-depletion enhances histone mobility at the nuclear interior. **a** Representative FRAP images for H2A-mCherry in control (siLacZ) cells, and upon single and co-depletion of Lamin A/C and Emerin. Scale bar ~ 10 μm. **b** (left) Mean % mobile fractions for internal and peripheral ROIs for H2A-mCherry in Control (siLac Z cells), single and co-depletion of Lamin A/C and Emerin. Data shown is a compilation from N = 3, 3–6 nuclei per replicate, Error bars = SEM, *p < 0.05, (right) Zoomed in image shows ROI (internal and peripheral) used for FRAP. **c-d** FRAP recovery curves for (**c**) peripheral and (**d**) internal ROIs for H2A-mCherry in control (siLacZ cells), single and co-depletion of Lamin A/C and Emerin. NFI = normalized fluorescence intensity, c (ii) and d (ii) rescaled graphs showing differences in recovery patterns between peripheral and internal ROIs. Data shown is representative experiment out of N = 3, 3–6 nuclei per replicate, Error bars: SEM. (N: number of independent biological replicates)

Since the co-depletion of Lamin A/C and Emerin alters chromosome territory positions (Figs. 4 and 5), we asked if Lamin A/C or Emerin depletion impacts chromatin dynamics. We performed Fluorescence Recovery After Photobleaching (FRAP) of fluorescently labelled Histone 2A (H2A) in living cells as a readout of chromatin dynamics [89] (Fig. 6). We simultaneously quantified recovery profiles of fluorescently tagged H2A (H2A-mCherry) at the nuclear interior and periphery, in order to sample chromatin dynamics at diverse regions of the nucleus (Fig. 6a, Table 3). Interestingly, mobile fractions of H2A-mCherry showed a significant increase at the nuclear interior in Lamin A/C-Emerin co-depleted cells (Mobile Fraction - M.F ~ 31%, Control - siLacZ: M.F ~ 16%) (Fig. 6b, d). In contrast, chromatin mobility at the nuclear interior or periphery was largely unaltered in single knockdowns of either Lamin A/C or Emerin (Fig. 6b-d). Taken together, these results suggest that Lamin A/C and Emerin co-depletion increases chromatin dynamics in the interphase nucleus.

**Table 3:** % Mobile fractions of H2A-mCherry upon co-depletion of Lamin A/C and Emerin

Experiment	Mean % Mobile Fraction (± SEM)	
	Internal	Peripheral
siLacZ	16.01 ± 1.48	14.12 ± 1.12
siLamin A/C	12.58 ± 2.32	13.58 ± 1.81
siEmerin	15.57 ± 2.61	13.80 ± 2.16
siLamin A/C + siEmerin	31.75 ± 5.46^a^ (*p* = 0.02381)	20.82 ± 3.71

^a^: statistically significant

### Rescue of chromatin mobility upon actin depolymerization and NM1 inhibition in Lamin A/C-Emerin co-depleted cells

**Fig. 7:**
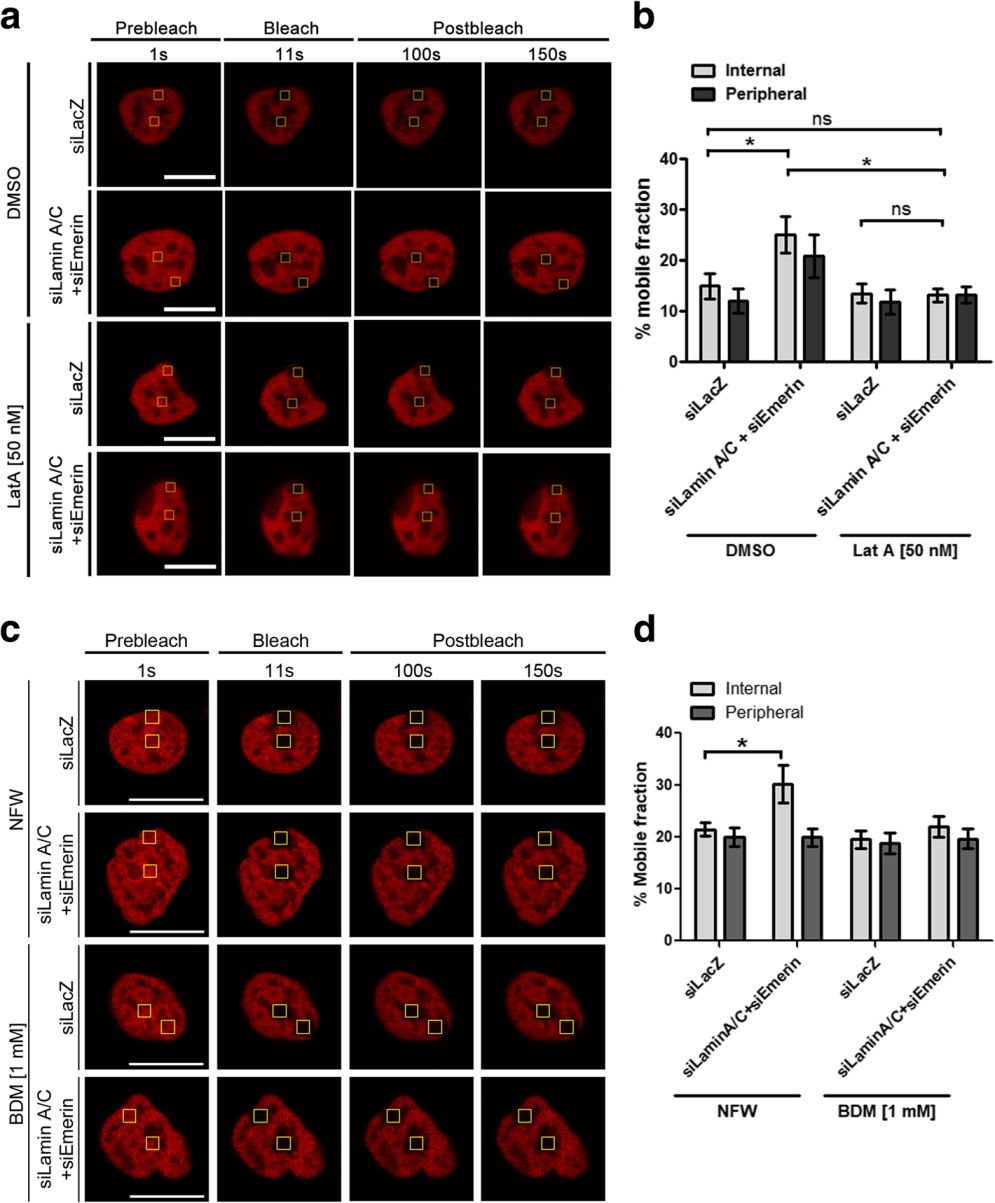
Actin depolymerization and NM1 inhibition in Lamin A/C-Emerin co-depleted cells restores chromatin dynamics. **a** FRAP images of H2A-mCherry in control (siLacZ) and siLamin A/C + siEmerin cells with DMSO (control) or Latrunculin A (50 nM) treatment. Scale bar ~ 10 μm. **b** Mean % mobile fractions for Internal and Peripheral ROIs for H2A-mCherry in Control (siLacZ) and siLamin A/C + siEmerin DLD-1 cells with DMSO or Latrunculin A (50 nM) treatment. Error bars: SEM, N = 3, ~ 6–12 nuclei per category, *p < 0.05. **c** FRAP images of H2A-mCherry in control (siLacZ) and siLamin A/C + siEmerin cells with NFW (control: nuclease free water) or BDM (1 mM) treatment. Scale bar ~ 10 μm. **d** Mean % mobile fractions for Internal and Peripheral ROIs for H2A-mCherry in Control (siLacZ) and siLamin A/C + siEmerin DLD-1 cells with NFW or BDM (1 mM) treatment. Error bars: SEM, N = 2, ~ 5–8 nuclei per category, *p < 0.05

The Lamin A/C-Emerin axis plays a crucial role in perceiving and relaying external mechanical signals into the nucleus and chromatin [59, 61, 85, 90–92]. Since we observed an impact of Lamin A/C-Emerin co-depletion on the subcellular distribution and organization of actin, we asked if destabilizing actin stress fibers impinges on chromatin mobility? We examined mobility of H2A-mCherry in Lamin A/C-Emerin co-depleted cells treated with Latrunculin A (Fig. 7a, Additional file 5a). Consistent with the previous experiment, combined knockdown of Lamin A/C and Emerin showed a significant increase in H2A-mCherry mobility toward the nuclear interior (siLamin A/C + siEmerin+DMSO: M.F ~ 25%, Control - siLacZ+DMSO: M.F ~ 14%) (Fig. 6b, 7b, Table 4). Remarkably, this enhanced mobility at the nuclear interior was restored to basal levels in Lamin A/C-Emerin co-depleted cells treated with Latrunculin A (M.F ~ 13%) (Fig. 7b, Table 4). H2A-mCherry mobility at the nuclear periphery was largely unaffected in Lamin A/C-Emerin co-depleted cells in the presence or absence of Latrunculin A (Lat A) treatment (Fig. 7b, Table 4). Chromatin mobility was also unaffected upon Lat A treatment alone, either at the nuclear interior or periphery respectively (Additional file 5b-c). In summary, depolymerization of actin in Lamin A/C-Emerin co-depleted cells restores chromatin mobility to basal levels, comparable to control cells (siLacZ).

**Table 4:** % Mobile fractions of H2A-mCherry upon Latrunculin A treatment of Lamin A/C and Emerin co-depleted cells

Experiment	Mean % Mobile Fraction (± SEM)	
	Internal	Peripheral
siLacZ + DMSO	14.94 ± 2.41	11.97 ± 2.44
siLamin A/C + siEmerin + DMSO	25.12 ± 3.60^a^ (*p* = 0.03717)	20.84 ± 4.12
siLacZ + Lat A [50 nM]	13.49 ± 1.85	11.81 ± 2.35
siLamin A/C + siEmerin + Lat A [50 nM]	13.14 ± 1.32	13.19 ± 1.60

^a^: statistically significant

We next asked if inhibiting NM1 activity impacts chromatin mobility in the interphase nucleus. We quantified the mobile fractions of H2A-mCherry in Lamin A/C-Emerin co-depleted cells upon inhibition of NM1 using BDM (Fig. 7c, Table 5). Histone mobility was significantly enhanced upon Lamin A/C-Emerin co-depletion as observed previously (siLamin A/C + siEmerin+NFW: M.F ~ 30%, Control - siLacZ+NFW: M.F ~ 21%), and was restored to basal levels in Lamin A/C-Emerin co-depleted cells treated with BDM – an inhibitor of NM1 activity (M.F ~ 22%) (Fig. 7d, Table 5). H2A-mCherry mobility at the nuclear periphery was largely unaffected in Lamin A/C-Emerin co-depleted cells upon BDM treatment (Fig. 7d, Table 5). Taken together, these results underscore a combined role of Lamin A/C and Emerin in the regulation of actin organization and NM1 activity, which further impinges on chromatin dynamics in the nucleus.

**Table 5:** % Mobile fractions of H2A-mCherry upon BDM treatment of Lamin A/C and Emerin co-depleted cells

Experiment	Mean % Mobile Fraction (± SEM)	
	Internal	Peripheral
siLacZ + NFW	21.48 ± 1.36	20.05 ± 1.79
siLamin A/C + siEmerin + NFW	30.17 ± 3.60^a^ (*p* = 0.04218)	19.92 ± 1.73
siLacZ + BDM [1 mM]	19.53 ± 1.73	18.78 ± 1.97
siLamin A/C + siEmerin + BDM [1 mM]	21.99 ± 1.99	19.64 ± 1.93

^a^: statistically significant

### Spatial positions of gene loci are altered upon Lamin A/C and Emerin depletion

**Fig. 8:**
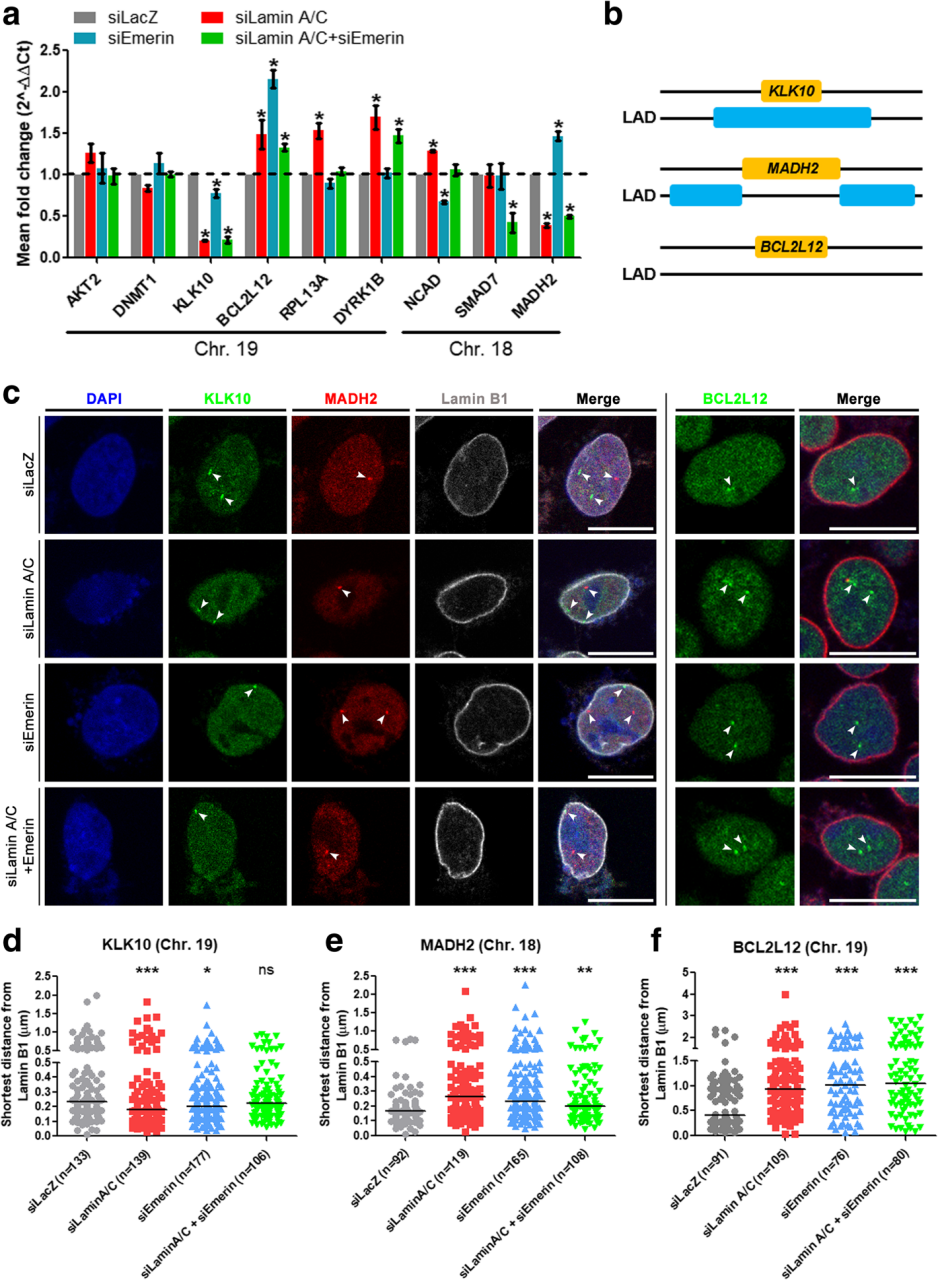
Spatial positions of gene loci are altered upon Lamin A/C and Emerin depletion. **a** qRT-PCR showing expression changes in candidate genes from chromosome 18 and 19 upon treatment of DLD-1 cells with siLacZ (control), siLamin A/C, siEmerin and siLamin A/C + siEmerin. Expression levels were first normalized to internal control GAPDH and then to siLacZ. Pooled data from N = 3 independent biological replicates, *p < 0.05. **b** Schematic representation of the Lamina Associated Domain (LAD) status of KLK10, SMAD2/MADH2 and BCL2L12. Blue bars represent the Lamin B1 Dam-ID data from Netherlands Cancer Institute (NKI). Snapshots from the UCSC Genome Browser have been provided in Additional files 6 and 7. **c** Representative mid-optical sections from confocal z- stacks of Immuno-3D FISH for KLK10 (Chr. 19), MADH2 (Chr. 18) and BCL2L12 (Chr. 19) gene loci in DLD-1 cells treated with siLacZ (control), siLamin A/C, siEmerin and siLamin A/C+Emerin. Nuclear envelope was demarcated by immunostaining for Lamin B1. **d** Shortest distance of KLK10 (Chr. 19) gene loci from Lamin B1 immunostaining in DLD-1 cells treated with siLacZ (control, Median (M), M = 0.233 μm), siLamin A/C (M = 0.181 μm), siEmerin (M = 0.201 μm) and siLamin A/C + siEmerin (M = 0.223 μm). **e** Shortest distance of MADH2 (Chr. 18) gene loci from Lamin B1 immunostaining in DLD-1 cells treated with siLacZ (control, M = 0.169 μm), siLamin A/C (M = 0.266 μm), siEmerin (M = 0.235 μm) and siLamin A/C + siEmerin (M = 0.199 μm). **f** Shortest distance of BCL2L12 (Chr. 19) gene loci from Lamin B1 immunostaining in DLD-1 cells treated with siLacZ (control, M = 0.413 μm), siLamin A/C (M = 0.929 μm), siEmerin (M = 1.02 μm) and siLamin A/C + siEmerin (M = 1.05 μm). **d**-**f**, Horizontal bar - Median (M): Pooled data from N = 2 independent biological replicates, *** *p* < 0.001, ** *p* < 0.01, * *p* < 0.05. Scale bar ~ 10 μm

Lamin A/C and Emerin regulate gene expression either directly or indirectly via other transcription factors [62, 93–102]. To examine the functional attributes of Lamin A/C and Emerin, we determined the expression levels of candidate genes (from human chromosomes 18 and 19), and their spatial localization in the interphase nucleus upon single and combined knockdowns of Lamin A/C and Emerin (Fig. 8a). Of the genes tested for expression changes, KLK10, BCL2L12 (both Chr. 19) and MADH2 (Chr. 18) showed transcriptional deregulation in both the single and co-depletions of Lamin A/C and Emerin (Fig. 8a). Interestingly, while KLK10 is within a Lamina Associated Domain (LAD), MADH2 is present between two neighboring LADs and BCL2L12 does not show LAD association or proximity (LAD-) (Fig. 8b, Additional files 6 a-h and 7a). We performed immuno-FISH assays to visualize the spatial localization of KLK10, BCL2L12 and MADH2 genes in the interphase nucleus, with reference to Lamin B1 staining as a marker of the nuclear envelope, as B-type lamins are unaffected upon Lamin A/C and Emerin knockdowns (Fig. 8c, Additional files 3 b and 7b). Measurement of the shortest distance from the nuclear envelope (Lamin B1 staining) revealed that KLK10 (LAD+), which was downregulated in both single and co-depletions of Lamin A/C and Emerin, was re-positioned closer to the nuclear envelope in single Lamin A/C and Emerin knockdowns (Fig. 8a, c-d). However, its location was unaffected upon Lamin A/C-Emerin co-depletion (Fig. 8d). In contrast, MADH2 (between two neighboring LADs), is downregulated in single Lamin A/C Kd, Lamin A/C-Emerin co-depletion and upregulated in single Emerin Kd (Fig. 8a). MADH2 gene loci were re-positioned away from the nuclear envelope upon both single and co-depletions of Lamin A/C and Emerin respectively (Fig. 8e). Additionally, BCL2L12 (LAD-) showed a significant movement away from the nuclear envelope, consistent with its upregulation upon single and co-depletions of Lamin A/C and Emerin (Fig. 8f). Taken together, these studies suggest that Lamin A/C and Emerin modulate gene expression levels as well as the spatial positions of gene loci in the interphase nucleus. Consistent with the differential localization and organization of Lamin A/C at the nuclear envelope and in the nucleoplasm, it is likely that different and potentially specific sub-interactomes of lamins differentially modulate chromosome territory positions and chromatin dynamics at the nuclear interior and the nuclear periphery. The Lamin A/C-Emerin-Actin-NM1 network is therefore a key axis required for the maintenance of genome organization and function in the nucleus.

## Discussion

The mechanisms that maintain chromosome position and function in the nucleus are largely unclear. Heterochromatin and gene poor chromosome territories are enriched in Lamina Associated Domains (LADs) and are localized toward the nuclear envelope [33, 39]. It is noteworthy that while B type lamins are largely associated with heterochromatin, Lamin A/C associates with both heterochromatin and euchromatin respectively [33, 103–105]. Here we show that the loss of Lamin A/C and Emerin significantly impacts subcellular localization of actin and NM1, which further impinges on chromatin organization (Figs. 2 and 3). Lamin A/C and Emerin regulate nuclear architecture via these interactors since actin depolymerization or NM1 inhibition restores enhanced chromatin dynamics to basal levels (Figs. 6 and 7).

### Lamin A/C and Emerin knockdown enhances chromatin dynamics

Lamin A/C predominantly exists as coiled-coil filaments beneath the inner nuclear membrane [106] and as a phosphorylated nucleoplasmic sub-population [107]. Emerin is localized in the cytoplasm and at the inner nuclear membrane, where it interacts with Lamin A/C [30]. Unlike Lamin A/C, Emerin does not exist as a freely diffusing nucleoplasmic sub-pool. Lamin A/C and Emerin form independent and interdependent interactomes in the nucleus [108]. The nucleoplasmic veil of Lamin A/C modulates chromatin organization at the nuclear interior, potentially via direct or indirect interactions with BANF1 and LEM-D proteins [109]. Lamin A/C in the nuclear interior constrains the mobility of an artificially integrated LacO gene locus, further suggesting a role for Lamin A/C in modulating chromatin dynamics [110]. An increase in Lamin A/C expression in differentiating stem cells correlates with restrained chromatin dynamics in the nucleus [11]. We speculate that the combined loss of Lamin A/C and Emerin destabilizes its interactors at the nuclear interior and envelope respectively. Chromatin at the nuclear periphery is associated with both A and B type Lamins, Emerin, Lamin B Receptor (LBR) and Lamina Associated Polypeptide 2β (LAP2β) among others [105, 111–113]. While euchromatin is associated with a nucleoplasmic sub-pool of Lamin A/C and interactors such as LAP2α [33, 103]. Notably, the localization of B type lamins was unaffected in Lamin A/C-Emerin co-depleted cells, highlighting a key role for Lamin A/C and Emerin in regulating chromatin dynamics (Additional file 3b). We surmise that Lamin A/C and Emerin co-depletion, potentially destabilizes its interactors at the nuclear interior, that therefore manifests as enhanced chromatin mobility at the interior as compared to the nuclear periphery (Fig. 6). Furthermore, locations of chromosome territories proximal to the nuclear envelope were unaffected, owing to the presence of B type Lamins (Additional file 3b), despite the loss of Lamin A/C and Emerin proteins at the nuclear envelope.

### Lamin A/C and Emerin modulate localization and activity of nuclear myosin 1

**Fig. 9:**
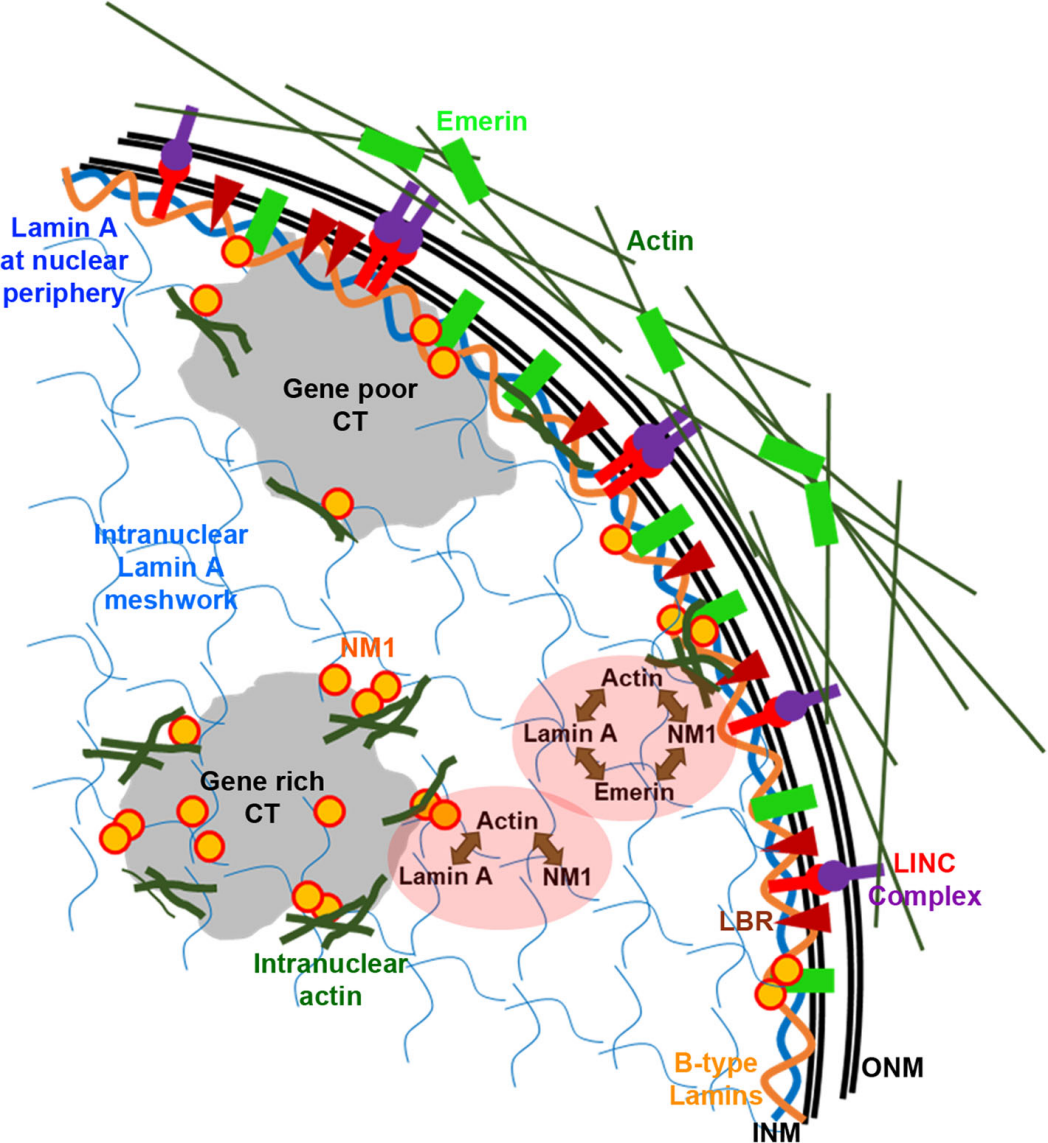
Schematic model suggesting a role for Lamin A/C and Emerin in regulating cytoskeletal and chromatin dynamics in the interphase nucleus. Lamin A/C and Emerin in association with actin and nuclear myosin (NM1), maintains cytoskeletal organization, which when destabilized, perturbs genome organization

NM1 is dependent on Lamin A/C and Emerin for its nuclear localization (Fig. 2). Major chromatin remodeling events during transcription and independent short and long-range chromatin movements are modulated by NM1 [114]. Although the interaction between NM1 and Emerin is well established [62], the functional relevance of such an interaction and its impact on chromatin organization remains unclear. Lamin A/C modulates the localization of NM1 in an Emerin dependent manner in the nucleus (Fig. 2). Moreover, NM1 associates with gene rich chromosome territories to a greater extent than gene poor chromosome territories [68, 80] suggesting an enhanced requirement for binding and activity of NM1 with gene rich chromosome territories. It is likely that NM1 activity is altered upon the depletion of Lamin A/C and Emerin, as the inhibition of NM1 activity via drug treatment (BDM), rescues enhanced chromatin mobility in Lamin A/C-Emerin co-depleted cells to basal levels (Fig. 7d). We speculate that Lamin A/C and Emerin co-regulate the sub-cellular organization and activity of NM1. Since NM1 is enriched on active chromatin, the loss of NM1 function is likely to impact chromatin preferentially near the nuclear interior than heterochromatin proximal to the nuclear envelope (Fig. 9).

### Actin reorganization modulates chromatin dynamics

Lamin A/C-Emerin co-depletion perturbs cytoskeletal organization as revealed by enhanced actin bundling, while Lamin A/C and Emerin knockdowns, redistribute NM1 foci into the nucleus (Figs. 2 and 3). Actin and NM1 are independently involved in modulating chromatin dynamics, required for long range chromatin movements. Reorganization of perinuclear F-actin reduces intranuclear G-actin [60]. RNA Polymerase II utilizes G actin for transcription [79]. We speculate that the reorganization of perinuclear F-actin upon Lamin A/C-Emerin co-depletion affects the organization of G-actin in the nucleus. This is consistent with a reduction in the levels of nuclear actin in Lamin A/C-Emerin co-depleted cells (Fig. 3c-e). Such a disruption of the actin network may primarily impact the activity and organization of euchromatin, since G-actin is required for remodeling transcriptionally active chromatin. Of note, enhanced chromatin mobility in the nuclear interior upon Lamin A/C and Emerin co-depletion was rescued upon Latrunculin A treatment, further underscoring the role of actin in modulating chromatin organization (Fig. 7b). Interestingly, perturbing actin organization affects the topology of chromosome territories and reduces gene loci dynamics at the nuclear interior rather than the periphery [115, 116]. This suggests the involvement of actin organization in maintaining chromatin organization and dynamics in the nucleus [116].

Taken together, we surmise that Lamin A/C and Emerin partner to modulate chromatin organization via actin and nuclear myosin 1. Surprisingly, the positions of gene poor chromosome territories proximal to the nuclear lamina were unaffected upon co-depletion of Lamin A/C and Emerin (Figs. 4 and 5). We speculate that while Lamin A/C-Emerin and their interactors distinctively regulate chromatin toward the nuclear interior, protein complexes associated with B type lamins such as LBR, HP1α among others, maintain chromatin organization closer to the nuclear periphery. Thus, Lamins and their sub-interactome maintain and fine-tune the spatial organization of chromosome territories in the interphase nucleus. Additionally, Lamin A/C and Emerin could also partner to maintain the organization and function of gene loci, since the loss of Lamin A/C or Emerin perturbs expression and localization of genes in the nucleus (Fig. 8). In summary, Emerin and Lamin A/C partner to maintain the spatio-functional organization of chromatin and its dynamics in the nucleus.

The functional significance of the mislocalization of gene rich chromosome territories upon Lamin A/C and Emerin depletion is unclear and remains to be investigated. Mislocalization of chromosome territories is likely to reorganize Topologically Associated Domains (TADs) and Lamina Associated Domains (LADs) [33, 117, 118]. Hi-C analyses might reveal the nature of chromatin contacts regulated by Lamin A/C-Emerin and the NM1-Actin sub-complex. An integrated study of chromatin conformation and its correlation with gene expression through single cell transcriptomics and high resolution imaging of single cells, may unravel the mechanistic underpinnings of how the cytoskeleton and the nucleus communicate with one another to enrich for specific and unique chromatin conformations, that in turn manifest into context specific transcriptional outputs, in a cell type and tissue specific manner.

## Conclusions

In conclusion, these studies reveal that Lamin A/C and Emerin partner to modulate genome organization via their cytoskeletal interactors – actin and NM1. Co-depletion of Lamin A/C and Emerin increases cytoplasmic actin stress fibers, decreases nuclear actin levels and potentially alters NM1 activity in the nucleus. This impacts genome organization as Lamin A/C and Emerin co-depletion enhances chromatin mobility toward the nuclear interior which was restored to basal levels upon NM1 inhibition or actin depolymerization. Impact on the genome was further reiterated by the mislocalization of chromosome territories upon Lamin A/C-Emerin co-depletion, and deregulation of gene expression levels of candidate genes - KLK10, BCL2L12 and MADH2, that differentially associate with the nuclear envelope, largely consistent with their Lamina Associated Domain (LAD) status. This study reinforces the intricate crosstalk between the cytoskeleton and the nucleus, orchestrated by Lamin A/C and Emerin that modulate cytoskeletal factors – actin and NM1.

## Methods

### Cell culture

DLD-1 colorectal adenocarcinoma cells were cultured in RPMI 1640 with 10% Fetal Bovine Serum, Penicillin (100 U/mL) and Streptomycin (100 μg/mL) (Invitrogen, Thermo Fischer Scientific, MA, USA) and were a gift from Dr. Thomas Ried, NCI/NIH, Bethesda, USA. Cells were routinely karyotyped to ascertain chromosomal stability and modal number of 44–46 chromosomes across multiple independent passages (Additional file 3a). Experiments were typically confined to cells cultured within the first 8–15 passages.

### siRNA transfection

siRNAs used for Lamin A/C and Emerin are siLMNA/C: 5′-CAGUCUGCUGAGAGGAACA-3′, siEMD1: 5′-UCUGACUUGAAUUCGACUA-3′ and siEMD2: 5′-UCCCAGAUGCUGACGCUUU-3′, non-targeting siRNA control siLACZ: 5′-CGUACGCGGAAUACUUCGA-3′. siRNA transfection mix was prepared using Lipofectamine RNAimax in Opti-MEM (Invitrogen, 13,778,150 and 31,985–070 respectively), ~ 0.2 X 10^6^ cells were plated with siRNA transfection mix for EMD (EMD1 and EMD2 siRNA oligos at 50 nM each) on day 1. Cells were pulsed with EMD1 and EMD2 oligos (50 nM each) for single EMD knockdown (Kd) and EMD1 + EMD2 (50 nM each) + LMNA/C (100 nM) for co-depletion (day 2). Single LMNA Kd was set up with LMNA/C siRNA (100 nM) (day 2). Knockdown continued for 48 h, followed by a change of media (day 4). Cells were harvested after 24 h (day 5).

### Western blotting

DLD-1 cells were lysed with Radio Immuno Precipitation Assay (RIPA) Buffer. Heat denatured protein samples were resolved on 10% acrylamide-bisacrylamide gel, transferred to an activated PVDF membrane (90 V for 90 min), blocked in 5% non-fat dried milk - 1 h/RT and incubated in primary antibodies prepared in 0.5% milk in 1X TBST. Primary antibodies used are as follows - anti- Lamin A (ab26300, 1:1000), anti-Lamin A/C (Jol2 ab40567, 1:200), anti-Actin (ab3280, 1:400), anti-Emerin (06–1052, 1:3000), anti-Emerin (ab40688, 1:1500), anti-MYO1C (ab51261, 1:1000) and anti-GAPDH (G9545, 1:5000). Secondary antibodies - anti-mouse IgG-HRP (GE, IL, USA- NA9310V, 1:5000) and anti-rabbit IgG-HRP (GE, IL, USA- NA9340V, 1:10000). Blots were incubated in primary antibody overnight at 4 °C and secondary antibodies for 1 h at RT respectively. Chemiluminescent substrates were used to develop the blots (GE ECL Prime (89168–782)).

### Immunofluorescence assay

DLD-1 cells were seeded on coverslips and siRNA or drug treatments were performed. Cells were washed with 1X Phosphate Buffered Saline (PBS) (2 X 5 min, RT) and fixed in 4% Paraformaldehyde (PFA) (in 1X PBS (pH 7.4)) for 10 min (RT), followed by two washes in 1X PBS washes. Cells were permeabilized in 0.5% Triton-X-100 (in 1X PBS) - 10 min, followed by 1X PBS washes (5 min, twice). Immunostaining protocol was followed as per Ranade et al. [86]. Primary antibodies used are as follows - anti-Lamin A (ab26300, 1:750), anti-Lamin A/C (Jol2 ab40567, 1:50), anti-Emerin (ab14208, 1:250), anti-Emerin (sc-25284, 1:500), anti-MYO1C (ab51261, 1:75) and anti-Lamin B2 (ab8983, 1:400). Secondary antibodies used are as follows - anti-Rabbit Alexa 488 (A11034, 1:1000) and anti-Mouse Alexa 568 (A11004, 1:1000).

### 3-dimensional fluorescence in situ hybridization (3D-FISH)

***Fixation*** 3D-FISH was performed as per standard protocols [86]. ***Hybridization*** Chromosome painting probes (Applied Spectral Imaging (ASI), Israel) were incubated at 37 °C for 5 min, denatured at 80 °C for 5 min, quick chilled on ice for 2 min and pre-annealed at 37 °C (~ 45 min). ~ 3–4 μl probe was applied to fixed cells on an 18 X 18 mm^2^ coverslip, co-denatured at 80 °C for 5 min, and hybridized for 48 h at 37 °C. ***Detection*** Coverslips were washed in 50% FA/2X SSC (pH 7.4) - 3 X 5 min at 45 °C, 0.1X SSC - 3 X 5 min at 60 °C, rinsed in 0.1% Tween20/4X SSC, counterstained with DAPI (3 min at RT), gently washed in 2X SSC and mounted. ***Imaging*** Image acquisition was performed on a Zeiss LSM 710 confocal microscope (Carl Zeiss, Thornwood, NJ, USA) or Leica TCS SP8 confocal laser scanning microscope (Leica Microsystems, Wetzlar, Germany) with 63X Plan-Apochromat 1.4 NA oil immersion objective. Image acquisition: zoom factor 2.0, Z-stacked images of voxel size 0.132 μm X 0.132 μm X 0.34 μm, 512X512 pixels per frame using 8-bit pixel depth for each channel. Line averaging: 2.0 and images acquired sequentially in a three-channel mode.

### Radial distance measurements of chromosome territories

3D radial distance measurements were performed using Image-Pro Plus (v 7.1). Single nuclei were cropped and subjected to 3D surface rendering followed by radial distance measurements of chromosome territories [86, 87].

### Statistical analysis

Statistical analysis was performed using GraphPad Prism 5.0 and Microsoft Excel. Radial Distances (% R.D) of chromosome territories and distances of gene loci were compared using Mann Whitney sum-rank test. Comparisons between proportions of cells showing NM1 mislocalization and actin stress fiber aggregates were done using Fisher’s exact test. Average mobile fractions were compared using unpaired Student’s-t-test. The non-targeting control (siLacZ) served as a reference for analyses of each data set. A *p*-value < 0.05 was considered as statistically significant.

### Fluorescence recovery after Photobleaching (FRAP) and analyses

Cells were grown on collagen (100 μg/ml) coated glass coverslips (22 X 22 mm^2^) placed on 35 mm petri dishes. Cells were transfected with H2A-mCherry construct (gift from Michael Davidson [Addgene plasmid # 55054]) for 24 h prior to photobleaching experiments. Cells were maintained in CO_2_ independent Leibovitz L-15 medium (Gibco, 21083–027) during photobleaching. Images were acquired using a 63X oil immersion objective with NA 1.4, 3X digital zoom in a Zeiss LSM 710 confocal microscope. Two independent Regions of Interest (ROI) of 20px X 20px (1 pixel = 0.0879 μm) were used for bleaching - one ROI in the nuclear interior and other near the nuclear border. Photobleaching conditions - 561 nm laser line, 80% laser power for photobleaching (100 iterations), 10 scans of pre-bleach, image acquisition every 1 s. Post bleach acquisition was performed in the duration when the relative nuclear motion and displacement was minimum. Images were analyzed using Zen 2011 FRAP Analysis module and Normalized Fluorescence Intensity (NFI) was calculated as follows:

$$ NFI=\frac{ROI1(t)- ROI3(t)}{ROI2(t)- ROI3(t)}\ X\frac{ROI2\left(t=0\right)- ROI3\left(t=0\right)}{ROI1\left(t=0\right)- ROI3\left(t=0\right)} $$

ROI1: fluorescence intensity - interior/peripheral ROI, ROI2: fluorescence intensity of a 20px X 20px region of the nucleus that was not subjected to photobleaching, and ROI3: fluorescence intensity of a 20px X 20px background region outside the nucleus. ROI1 (t) denotes the post-bleach fluorescence intensity at time t. ROI2 (t) and ROI3 (t) denote the same for unbleached ROI and background, respectively. ROI1 (t = 0) denotes the average pre-bleach fluorescence intensity. ROI2 (t = 0) and ROI3 (t = 0) denote the same for unbleached ROI and background, respectively. The NFI was plotted as a function of time to generate double normalized FRAP curves. Mobile fractions of H2A-mCherry were calculated as follows:

$$ \% Mobile\ fraction=\frac{Ffinal- Fbleach}{Fprebleach- Fbleach} \times 100 $$

Where, Ffinal is the NFI at maximum recovery (saturation), Fbleach is the NFI at the instant of bleaching and Fpre-bleach is the NFI before bleaching [119].

### Actin depolymerization and NM1 inhibition for live imaging

siRNA mediated co-depletion of Lamin A/C and Emerin was performed as mentioned previously, and H2A-mCherry overexpression was carried out 24 h prior to photobleaching experiments. Leibovitz L-15 medium was added to the Lamin A/C-Emerin co-depleted cells (and siLacZ control cells), followed by treatment with either 50 nM Latrunculin A or 1 mM BDM for 60 mins. FRAP and live imaging of cells was performed in the presence of the inhibitor for the next 45–60 min.

### Co-immunoprecipitation

Co-immunoprecipitation was performed using Co-IP Lysis Buffer (50 mM Tris pH 8.0, 300 mM NaCl, 0.5% NP-40, 1X Protease Inhibitor Cocktail - Roche, Basel, Switzerland). Cells were lysed in Co-IP Lysis Buffer, incubated on ice (30 min) and centrifuged at 14000 rpm for 15 min at 4 °C. For Co-IP, 120 μg of lysate (each from Control and siLamin A/C cells) was pre-cleared using Protein G/A-Dynabeads (45 min at 4 °C). Primary antibody (anti-Emerin sc25284, anti-MYO1C ab51261) (1 μg) and normal mouse/rabbit IgG (1 μg) were added, followed by overnight incubation at 4 °C. Antigen-antibody complexes were captured (4 h at 4 °C) using 20 μl Protein G/A-Dynabeads (blocked previously with 0.5 mg/ml BSA in 1X PBS [30 min/4 °C]). Beads were washed six times with Co-IP Lysis buffer, boiled in 4X Laemmli, resolved on polyacrylamide gel and subjected to western blotting.

### Preparation of nuclear and cytoplasmic extracts

REAP Protocol was followed [120].

### Immuno-3D FISH

***Fixation for 3-D ImmunoFISH*** DLD-1 cells growing on coverslips and subjected to Lamin A/C and Emerin knockdowns were treated with CSK buffer for 6.5 mins, followed by fixation using 4% PFA (pH = 7.2, PFA, Sigma, 158,127) for 12 mins at RT. After two washes in 1X PBS, cells were permeabilized in 0.5% Triton X-100 (in 1X PBS) for 15 mins, followed by incubation in 20% glycerol (in 1X PBS) for 45 mins. Cells were subjected to 5 freeze-thaw cycles in liquid nitrogen, followed by 3 washes in 1X PBS. Cells were denatured in 0.1 N HCl for 10 mins, followed by two washes in 50% FA-2X SSC (pH 7.4). Nuclei were stored in 50% FA-2X SSC (pH 7.4) at 4 °C overnight or until further use. ***Immunofluorescence Assay*** Cells fixed previously on coverslips were washed briefly using 1X PBS (5 min, once at RT). Blocking was performed in 1% BSA (Sigma, A2153) solution for 30 mins. The primary antibody Rabbit anti-Lamin B1 (ab16048, 1:1000) was diluted in 0.5% BSA and cells were incubated at RT for 90 min. Secondary antibody Goat anti-Rabbit Alexa-633 (A21070, 1:1000) diluted in 1X PBS-Triton X-100 (1X PBST) was applied to cells on coverslips at RT for 60 min. ***Hybridization and post-hybridization washes*** After the final 3 washes of 1X PBST, coverslips were stored in 1X PBST for 45 mins. Post-fixation was in 4% PFA for 7 mins and post-permeabilization in 0.5% Triton X-100 for 7 mins, followed by 2 washes in 1X PBS and 2 washes in 50% FA-2X SSC. BAC clone RP11-380 M21 for MADH2, RP11-26P12 for KLK10 and RP11-264 M8 for BCL2L12 were purified using isolation protocol by Villalobos et al., optimized for 100 ml cultures [121]. Required amount of MADH2, KLK10 and BCL2L12 probe labelled with Texas Red and Rhodamine dUTPs respectively using Nick Translation Kit (Roche, following kit protocol) was incubated at 37 °C for 7 mins/750 rpm. Probe was denatured at 80 °C for 5 mins and quick chilled on ice for 2 mins. Pre-annealing was at 37 °C for 45 mins. Co-denaturation of MADH2, KLK10 and BCL2L12 probe and immunostained nuclei was at 80 °C for 7 mins, followed by hybridization in a moist sealed chamber at 37 °C for 48 h. Post hybridization washes were with 50% FA-2X SSC at 45 °C (3 washes/5 mins each) and 0.1X SSC at 60 °C (3 washes/5 mins each). Nuclei were counterstained with DAPI and mounted in Antifade. ***Imaging*** Image acquisition was performed on a Zeiss LSM 710 confocal microscope (Carl Zeiss, Thornwood, NJ, USA) with 63X Plan-Apochromat 1.4 NA oil immersion objective. Image acquisition: zoom factor 1.5, Z-stacked images of voxel size 0.18 μm X 0.18 μm X 0.34 μm, 512 X 512 pixels per frame using 8-bit pixel depth for each channel. Line averaging: 4.0 and images acquired sequentially in a three-channel mode.

## Additional files

Additional file 1: (a) Immunostaining for Emerin (green), Lamin A/C (red) in untreated, siLamin A/C and siLamin A/C scrambled treated DLD-1 cells at the end of 48 h post Lamin A/C Kd. Scale bar ~ 10 μm. (b) Quantification of number of cells showing mislocalization of Emerin as an extranuclear aggregate in control and Lamin A/C kd cells, n = number of cells analysed, compiled data from *N* = 2 independent biological replicates. (c) Representative mid-optical section from confocal z-stacks of NM1 immunostaining in siLacZ, siLamin A/C, siEmerin and siLamin A/C + siEmerin treated DLD-1 cells, showing the localization of NM1 foci inside the nucleus. Scale bar ~ 10 μm. (TIF 3756 kb)

Additional file 2: (a) Representative western blot showing expression levels of NM1 in single and combined depletion of Lamin A/C and Emerin in DLD-1 cells. (b) Densitometric quantification of band intensities for NM1 in single and combined depletion of Lamin A/C and Emerin. Error bar: SEM, compiled data from *N* = 5 independent biological replicates. (c) Above: Co-IP using anti-Emerin antibody, probed for interaction with NM1 in control (siLacZ) and Lamin A/C depleted cells. Below: Western blot showing Lamin A/C depletion. Loading control: Actin (d) Densitometric quantification of band intensities for NM1 in Emerin pulldown upon Lamin A/C depletion. Both Emerin and NM1 levels were normalized to their respective inputs and the extent of NM1 pulled down with Emerin was further normalized to total Emerin pulldown. Error bar: SEM, compiled data from *N* = 3 independent biological replicates. (TIF 1956 kb)

Additional file 3: (a) Metaphase counts of DLD-1 cells consistently show pseudo-diploid chromosome numbers of 44–46. Scale bar ~ 10 μm. (b) Immunostaining for Lamin B2 (red) upon Lamin A/C and Emerin co-depletion in DLD-1 cells. Scale bar ~ 10 μm. (c) Representative western blot showing Actin levels in whole cell extract of DLD-1 cells with single and combined depletion of Lamin A/C-Emerin. (d) Densitometric quantification of band intensities for Actin in whole cell extracts from cells with single and combined depletion of Lamin A/C-Emerin. Error bar: SEM, compiled data from N = 3 independent biological replicates, *p < 0.05. (TIF 3645 kb)

Additional file 4: (a-b) Dot scatter plots showing nuclear area (a) and volume (b) upon single and combined depletion of Lamin A/C and Emerin**.** Control - siLacZ treated cells, Horizontal bar: Median (M), n: number of nuclei, compiled data from N = 3 independent biological replicates. (c-d) Dot scatter plots showing CT18 area (c) and volume (d) upon single and combined depletion of Lamin A/C and Emerin. Control - siLacZ treated cells, Horizontal bar: Median (M), n: number of CTs, data compiled from *N* = 2 independent biological replicates. (e-f) Dot scatter plots showing CT19 area (e) and volume (f) upon single and combined depletion of Lamin A/C and Emerin**.** Control - siLacZ treated cells, Horizontal bar: Median (M), n: number of CTs, compiled data from N = 2 independent biological replicates. (g-h) Dot scatter plots showing CT7 area (g) and volume (h) upon single and combined depletion of Lamin A/C and Emerin**.** Control - Untreated cells (UT), Horizontal bar: Median (M), n: number of CTs, *N* = 1 (data from single experiment). (i-j) Dot scatter plots depicting CT17 area (i) and volume (j) upon single and combined depletion of Lamin A/C and Emerin**.** Control - Untreated cells (UT), Horizontal bar: Median (M), n: number of CTs, N = 1 (data from single experiment), *p < 0.05, **p < 0.01, ***p < 0.001. (TIF 3572 kb)

Additional file 5: (a) Phalloidin staining (green) performed on cells treated with 50 nM and 100 nM Latrunculin A for 90 min. DMSO treated and untreated cells were used as controls. Depolymerization of actin was ascertained using reduced phalloidin staining upon Lat A treatment. Scale bar ~ 10 μm. (b) FRAP performed on H2A-mCherry in control (DMSO) and Lat A (50 nM) treated DLD-1 cells. Two ROIs were used for the experiment – interior of the nucleus (Internal) and proximal to nuclear periphery (Peripheral), ~ 4–5 nuclei were assayed for recovery fractions in each condition per experiment. (c) Mean % mobile fraction for control (DMSO) and Lat A (50 nM) treated DLD-1 cells. No significant difference was detected for the mobile fractions of H2A-mCherry in either the intranuclear or peripheral pools upon Lat A treatment (50 nM, 90 min) (*p* > 0.05, Unpaired Student’s t-test). Error bars: SEM, compiled data from N = 2 independent biological replicates. (TIF 2609 kb)

Additional file 6: (a-h) UCSC Genome Browser view for (a) KLK10, (b) MADH2/SMAD2, (c) AKT2, (d) DNMT1, (e) DYRK1B, (f) RPL13A, (g) NCAD/CDH2 and (h) SMAD7, also depicting the Lamina Associated Domains (NKI LAD track). (TIFF 2608 kb)

Additional file 7: (a) UCSC Genome Browser view for BCL2L12, depicting the Lamina Associated Domains (NKI LAD track). (b) Representative mid-optical sections from confocal z-stacks of Immuno-3D FISH for BCL2L12 (Chr. 19) gene loci in DLD-1 cells treated with siLacZ (control), siLamin A/C, siEmerin and siLamin A/C + siEmerin. Nuclear envelope was demarcated using immunostaining of Lamin B1. Scale bar ~ 10 μm. (TIFF 2263 kb)

## Abbreviations

CT: Chromosome territory
LAD: Lamina Associated Domain
LEM-D: LEM(LAP2-Emerin-MAN1)-domain
RD: Radial distance
