# Supplementary figures and images for: Lamin A/C and Emerin depletion impacts chromatin organization and dynamics in the interphase nucleus
Source: BMC Mol Cell Biol. 2019 May 22;20:11. doi: 10.1186/s12860-019-0192-5 (PMC6532135; doi:10.1186/s12860-019-0192-5)

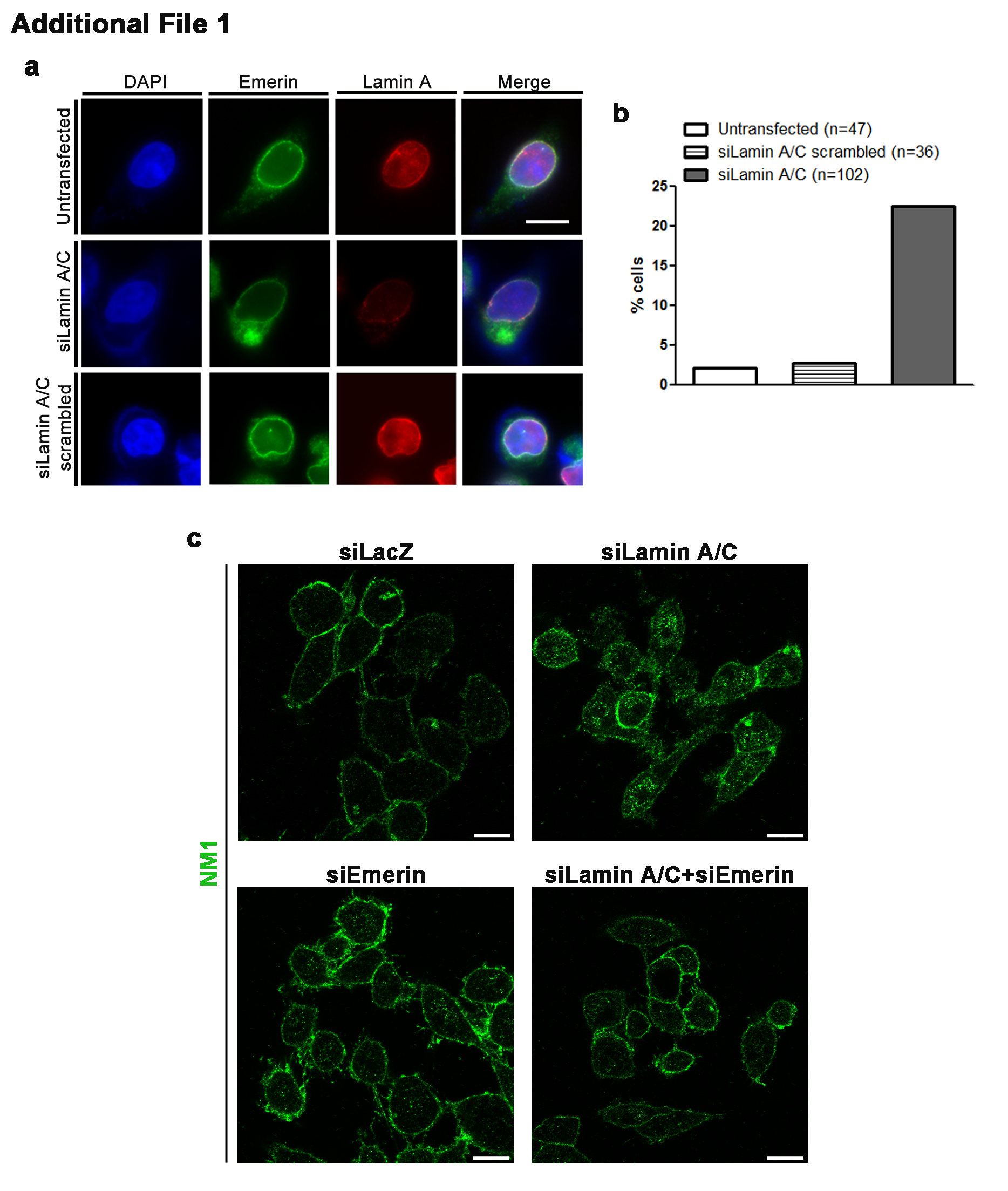

Supplement: Supplementary file 1 — (a) Immunostaining for Emerin (green), Lamin A/C (red) in untreated, siLamin A/C and siLamin A/C scrambled treated DLD-1 cells at the end of 48 h post Lamin A/C Kd. Scale bar ~ 10 μm. (b) Quantification of number of cells showing mislocalization of Emerin as an extranuclear aggregate in control and Lamin A/C kd cells, n = number of cells analysed, compiled data from N = 2 independent biological replicates. (c) Representative mid-optical section from confocal z-stacks of NM1 immunostaining in siLacZ, siLamin A/C, siEmerin and siLamin A/C + siEmerin treated DLD-1 cells, showing the localization of NM1 foci inside the nucleus. Scale bar ~ 10 μm. (TIF 3756 kb) [file 12860_2019_192_MOESM1_ESM.tif]

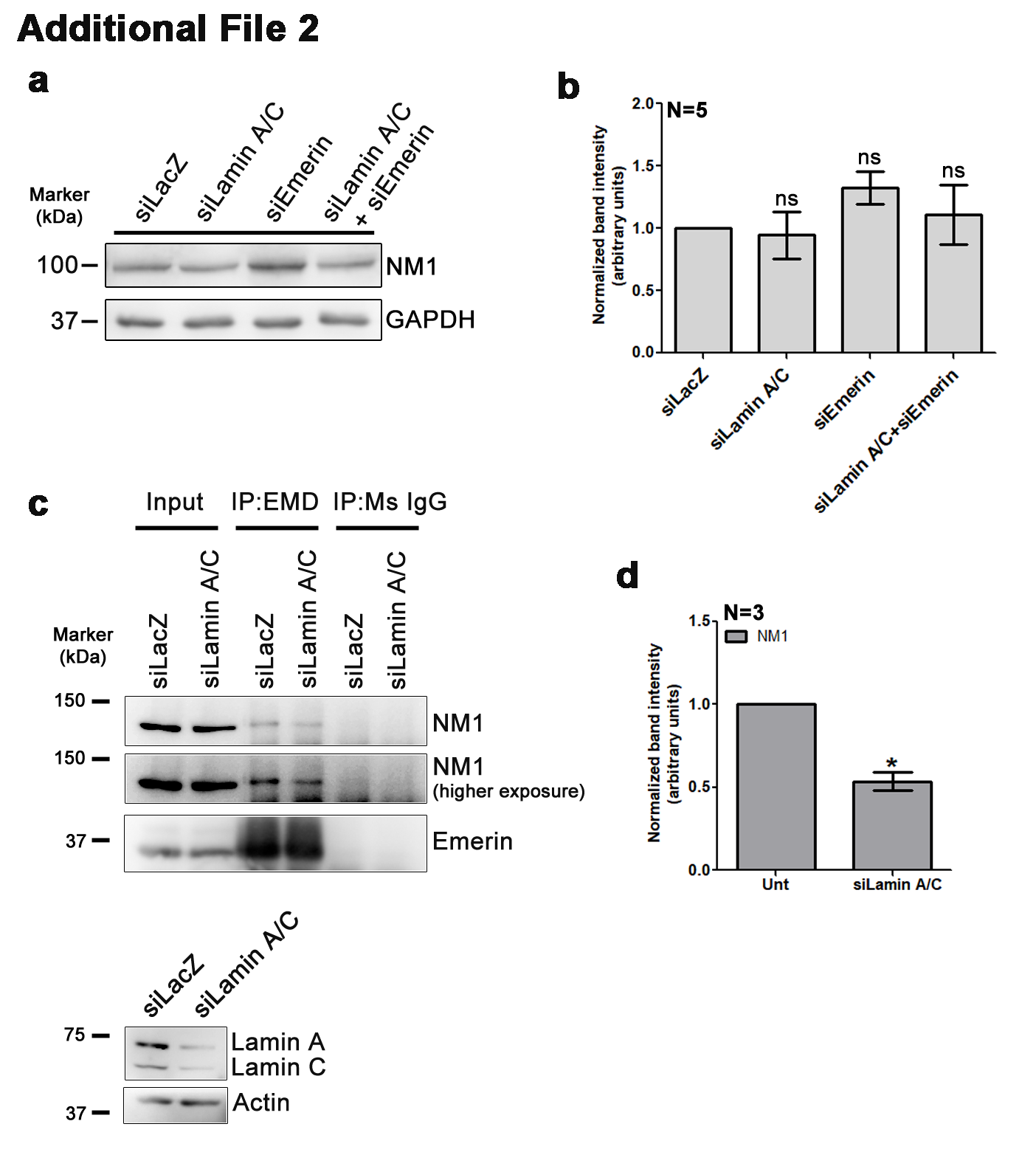

Supplement: Supplementary file 2 — (a) Representative western blot showing expression levels of NM1 in single and combined depletion of Lamin A/C and Emerin in DLD-1 cells. (b) Densitometric quantification of band intensities for NM1 in single and combined depletion of Lamin A/C and Emerin. Error bar: SEM, compiled data from N = 5 independent biological replicates. (c) Above: Co-IP using anti-Emerin antibody, probed for interaction with NM1 in control (siLacZ) and Lamin A/C depleted cells. Below: Western blot showing Lamin A/C depletion. Loading control: Actin (d) Densitometric quantification of band intensities for NM1 in Emerin pulldown upon Lamin A/C depletion. Both Emerin and NM1 levels were normalized to their respective inputs and the extent of NM1 pulled down with Emerin was further normalized to total Emerin pulldown. Error bar: SEM, compiled data from N = 3 independent biological replicates. (TIF 1956 kb) [file 12860_2019_192_MOESM2_ESM.tif]

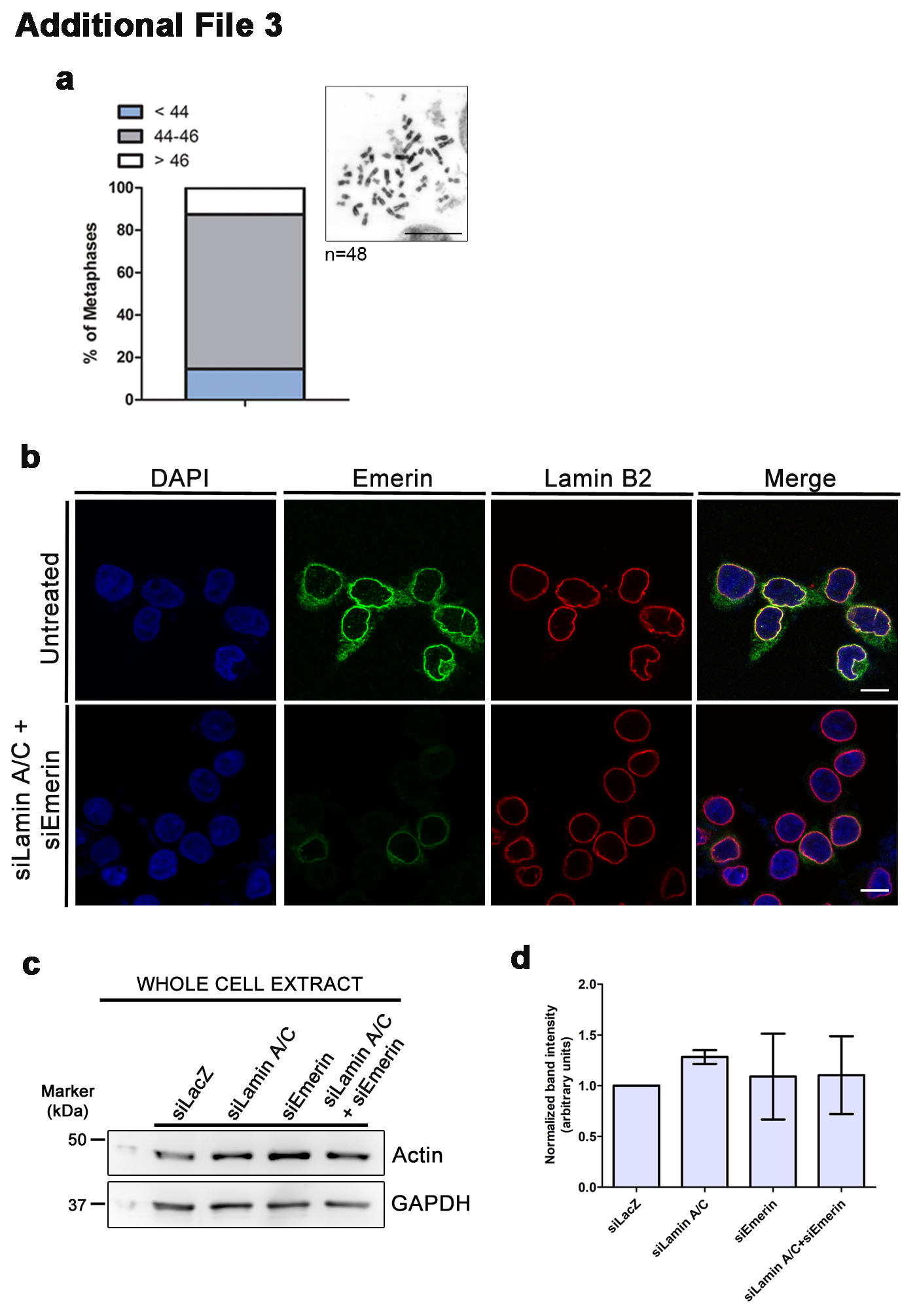

Supplement: Supplementary file 3 — (a) Metaphase counts of DLD-1 cells consistently show pseudo-diploid chromosome numbers of 44–46. Scale bar ~ 10 μm. (b) Immunostaining for Lamin B2 (red) upon Lamin A/C and Emerin co-depletion in DLD-1 cells. Scale bar ~ 10 μm. (c) Representative western blot showing Actin levels in whole cell extract of DLD-1 cells with single and combined depletion of Lamin A/C-Emerin. (d) Densitometric quantification of band intensities for Actin in whole cell extracts from cells with single and combined depletion of Lamin A/C-Emerin. Error bar: SEM, compiled data from N = 3 independent biological replicates, *p < 0.05. (TIF 3645 kb) [file 12860_2019_192_MOESM3_ESM.tif]

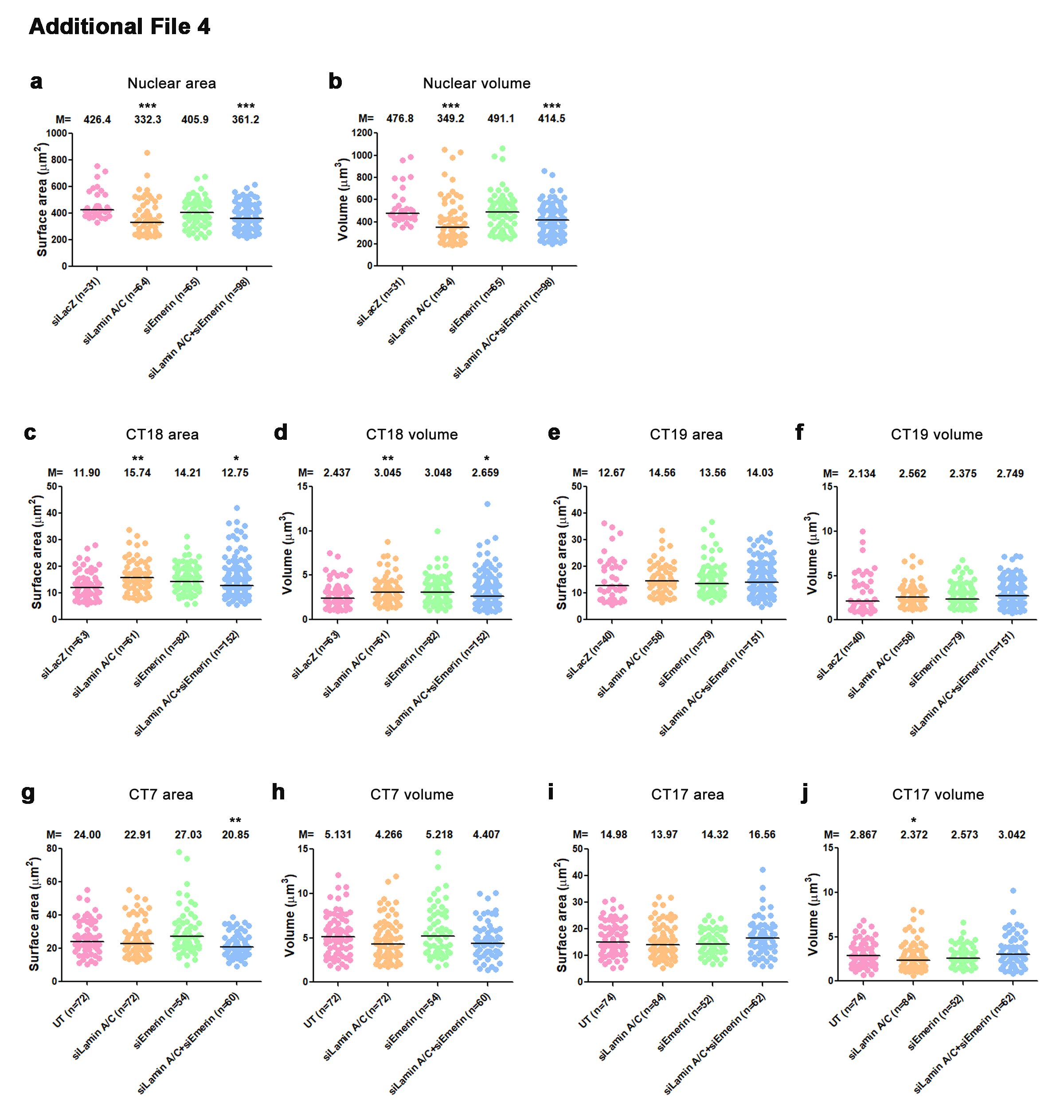

Supplement: Supplementary file 4 — (a-b) Dot scatter plots showing nuclear area (a) and volume (b) upon single and combined depletion of Lamin A/C and Emerin. Control - siLacZ treated cells, Horizontal bar: Median (M), n: number of nuclei, compiled data from N = 3 independent biological replicates. (c-d) Dot scatter plots showing CT18 area (c) and volume (d) upon single and combined depletion of Lamin A/C and Emerin. Control - siLacZ treated cells, Horizontal bar: Median (M), n: number of CTs, data compiled from N = 2 independent biological replicates. (e-f) Dot scatter plots showing CT19 area (e) and volume (f) upon single and combined depletion of Lamin A/C and Emerin. Control - siLacZ treated cells, Horizontal bar: Median (M), n: number of CTs, compiled data from N = 2 independent biological replicates. (g-h) Dot scatter plots showing CT7 area (g) and volume (h) upon single and combined depletion of Lamin A/C and Emerin. Control - Untreated cells (UT), Horizontal bar: Median (M), n: number of CTs, N = 1 (data from single experiment). (i-j) Dot scatter plots depicting CT17 area (i) and volume (j) upon single and combined depletion of Lamin A/C and Emerin. Control - Untreated cells (UT), Horizontal bar: Median (M), n: number of CTs, N = 1 (data from single experiment), *p < 0.05, **p < 0.01, ***p < 0.001. (TIF 3572 kb) [file 12860_2019_192_MOESM4_ESM.tif]

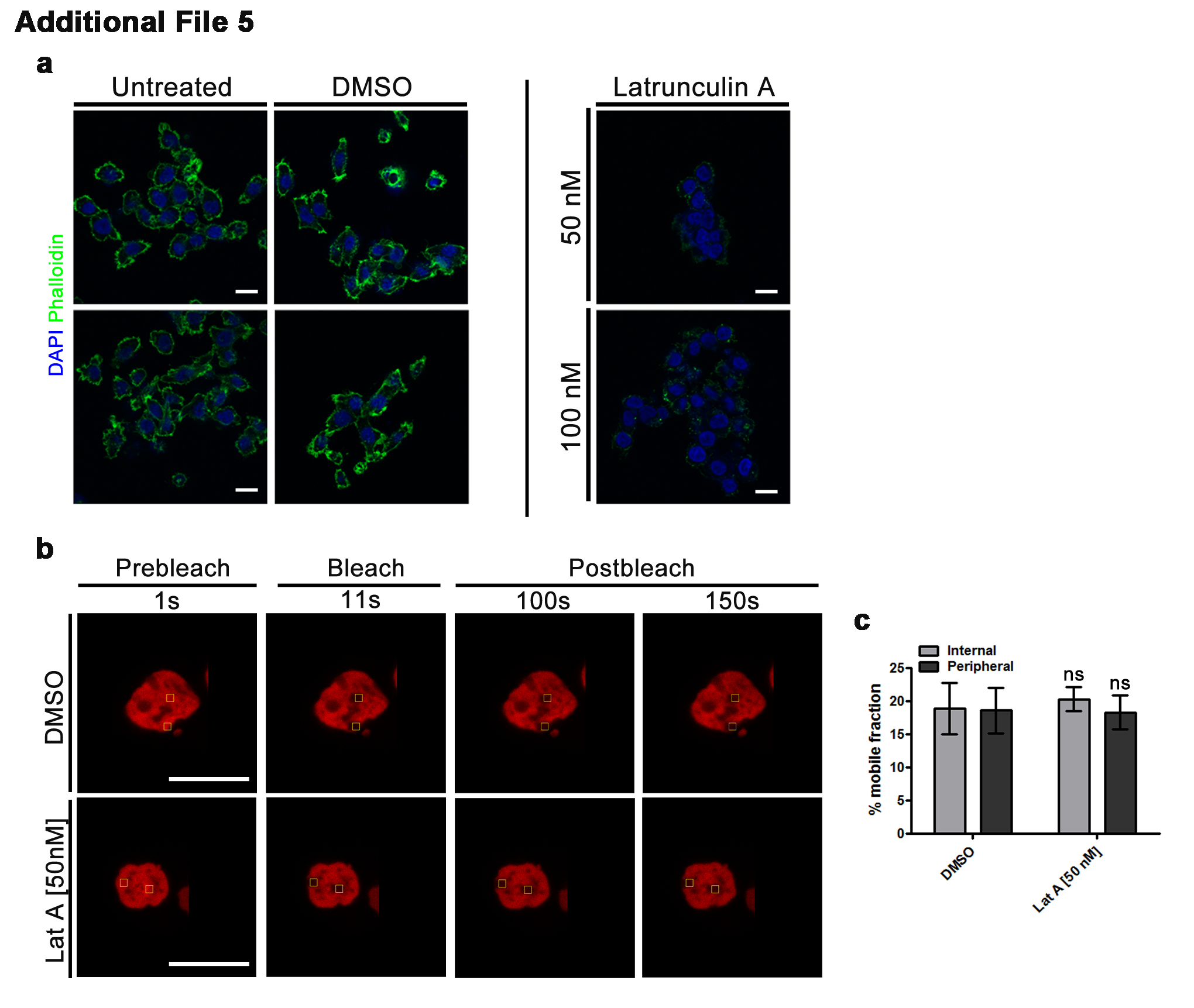

Supplement: Supplementary file 5 — (a) Phalloidin staining (green) performed on cells treated with 50 nM and 100 nM Latrunculin A for 90 min. DMSO treated and untreated cells were used as controls. Depolymerization of actin was ascertained using reduced phalloidin staining upon Lat A treatment. Scale bar ~ 10 μm. (b) FRAP performed on H2A-mCherry in control (DMSO) and Lat A (50 nM) treated DLD-1 cells. Two ROIs were used for the experiment – interior of the nucleus (Internal) and proximal to nuclear periphery (Peripheral), ~ 4–5 nuclei were assayed for recovery fractions in each condition per experiment. (c) Mean % mobile fraction for control (DMSO) and Lat A (50 nM) treated DLD-1 cells. No significant difference was detected for the mobile fractions of H2A-mCherry in either the intranuclear or peripheral pools upon Lat A treatment (50 nM, 90 min) (p > 0.05, Unpaired Student’s t-test). Error bars: SEM, compiled data from N = 2 independent biological replicates. (TIF 2609 kb) [file 12860_2019_192_MOESM5_ESM.tif]

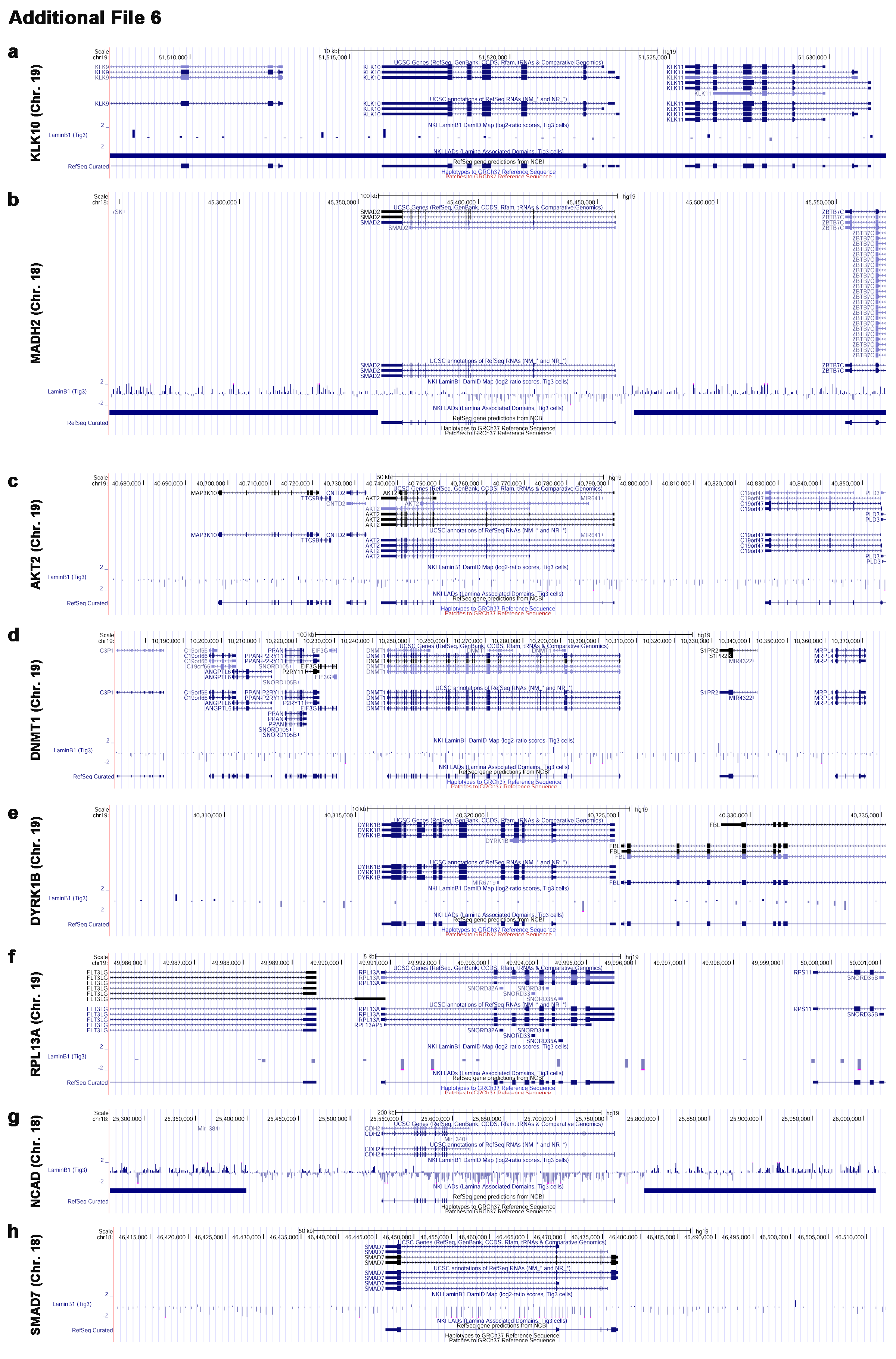

Supplement: Supplementary file 6 — (a-h) UCSC Genome Browser view for (a) KLK10, (b) MADH2/SMAD2, (c) AKT2, (d) DNMT1, (e) DYRK1B, (f) RPL13A, (g) NCAD/CDH2 and (h) SMAD7, also depicting the Lamina Associated Domains (NKI LAD track). (TIFF 2608 kb) [file 12860_2019_192_MOESM6_ESM.tiff]

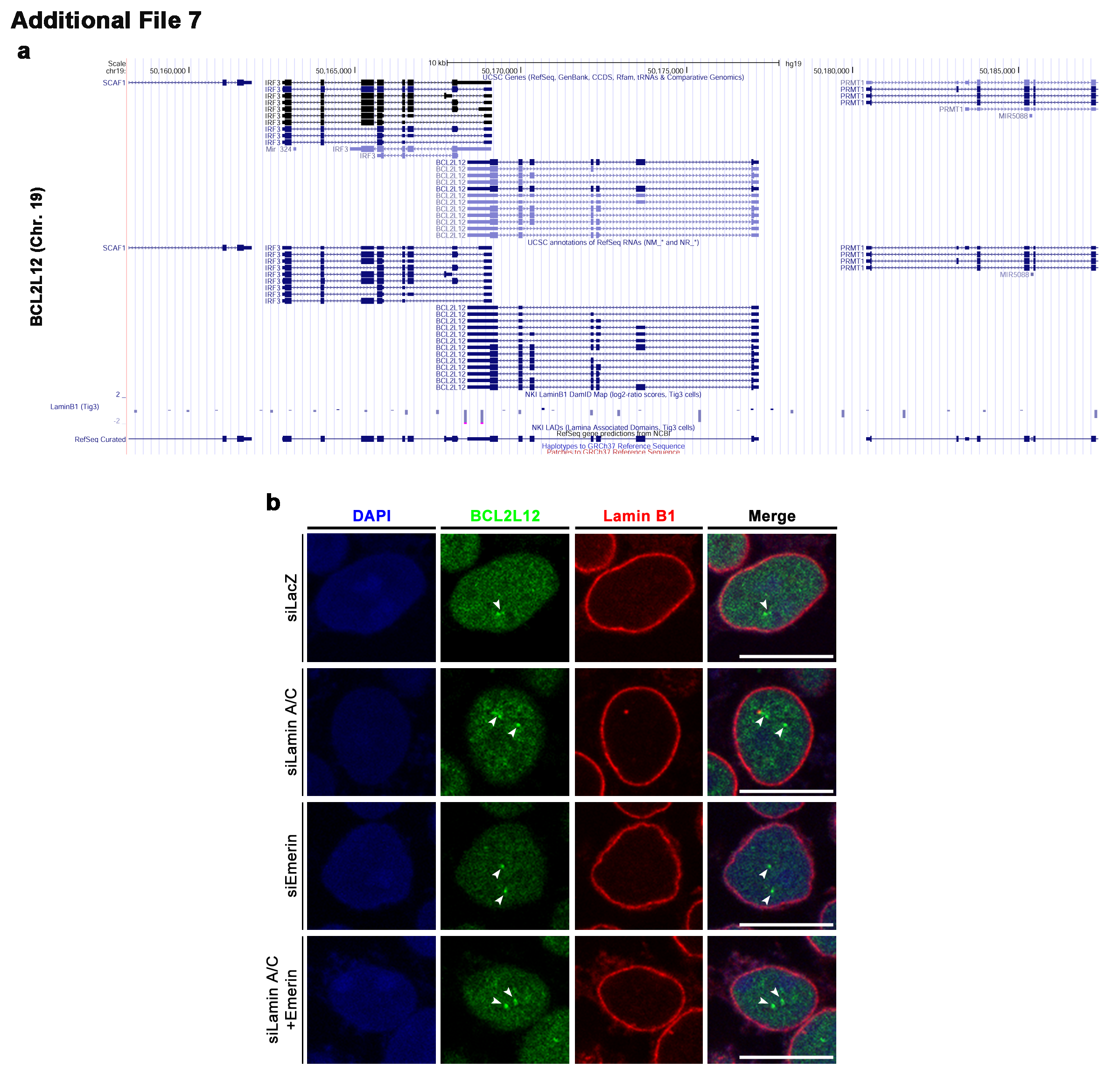

Supplement: Supplementary file 7 — (a) UCSC Genome Browser view for BCL2L12, depicting the Lamina Associated Domains (NKI LAD track). (b) Representative mid-optical sections from confocal z-stacks of Immuno-3D FISH for BCL2L12 (Chr. 19) gene loci in DLD-1 cells treated with siLacZ (control), siLamin A/C, siEmerin and siLamin A/C + siEmerin. Nuclear envelope was demarcated using immunostaining of Lamin B1. Scale bar ~ 10 μm. (TIFF 2263 kb) [file 12860_2019_192_MOESM7_ESM.tiff]
